# CD8^+^ T cell-intrinsic IL-6 signaling promotes resistance to anti-PD-L1 immunotherapy

**DOI:** 10.1016/j.xcrm.2022.100878

**Published:** 2023-01-03

**Authors:** Mahrukh A. Huseni, Lifen Wang, Joanna E. Klementowicz, Kobe Yuen, Beatrice Breart, Christine Orr, Li-fen Liu, Yijin Li, Vinita Gupta, Congfen Li, Deepali Rishipathak, Jing Peng, Yasin Şenbabaoǧlu, Zora Modrusan, Shilpa Keerthivasan, Shravan Madireddi, Ying-Jiun Chen, Eleanor J. Fraser, Ning Leng, Habib Hamidi, Hartmut Koeppen, James Ziai, Kenji Hashimoto, Marcella Fassò, Patrick Williams, David F. McDermott, Jonathan E. Rosenberg, Thomas Powles, Leisha A. Emens, Priti S. Hegde, Ira Mellman, Shannon J. Turley, Mark S. Wilson, Sanjeev Mariathasan, Luciana Molinero, Mark Merchant, Nathaniel R. West

**Affiliations:** 1 Genentech, South San Francisco, CA 94080, USA; 2 Beth Israel Deaconess Medical Center, Boston, MA 02215, USA; 3 Genitourinary Oncology Service, Department of Medicine, Memorial Sloan Kettering Cancer Center, New York, NY 10065, USA; 4 Barts Experimental Cancer Medicine Centre, Barts Cancer Institute, Queen Mary University of London, London EC1M 6BQ, UK; 5 University of Pittsburgh Medical Center, Hillman Cancer Center, Pittsburgh, PA 15213, USA

**Keywords:** checkpoint blockade immunotherapy, interleukin 6, IL-6, atezolizumab, PD-L1, CD8 T cell, clinical trial, cancer

## Abstract

Although immune checkpoint inhibitors (ICIs) are established as effective cancer therapies, overcoming therapeutic resistance remains a critical challenge. Here we identify interleukin 6 (IL-6) as a correlate of poor response to atezolizumab (anti-PD-L1) in large clinical trials of advanced kidney, breast, and bladder cancers. In pre-clinical models, combined blockade of PD-L1 and the IL-6 receptor (IL6R) causes synergistic regression of large established tumors and substantially improves anti-tumor CD8^+^ cytotoxic T lymphocyte (CTL) responses compared with anti-PD-L1 alone. Circulating CTLs from cancer patients with high plasma IL-6 display a repressed functional profile based on single-cell RNA sequencing, and IL-6-STAT3 signaling inhibits classical cytotoxic differentiation of CTLs *in vitro*. In tumor-bearing mice, CTL-specific IL6R deficiency is sufficient to improve anti-PD-L1 activity. Thus, based on both clinical and experimental evidence, agents targeting IL-6 signaling are plausible partners for combination with ICIs in cancer patients.

## Highlights

• High IL-6 associates with poor atezolizumab efficacy in patients with advanced cancer
• IL6R- and PD-L1-blocking antibodies combine synergistically to treat murine tumors
• IL-6-STAT3 signaling blocks cytotoxic effector differentiation of CD8^+^ T cells
• Cell-intrinsic IL-6 signaling in CD8^+^ T cells limits pre-clinical anti-PD-L1 activity

## Graphical abstract

**Figure:**
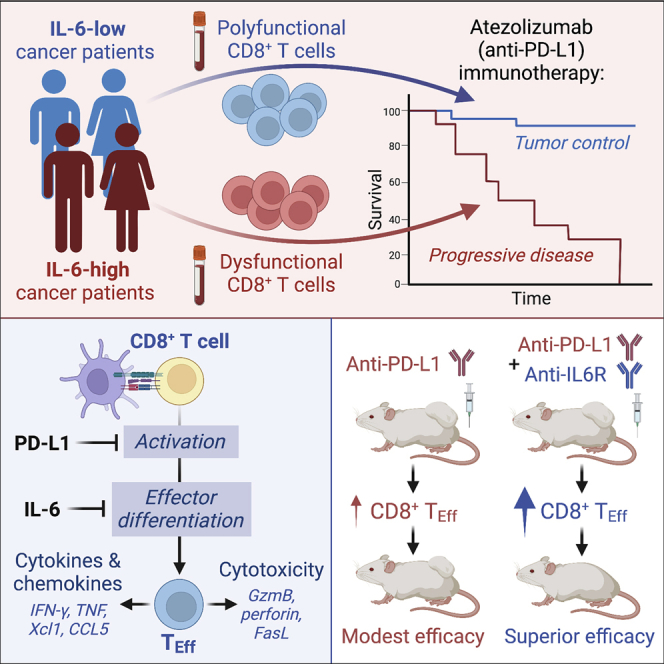

## Teaser

Identifying clinically actionable drivers of therapeutic resistance is a major objective for cancer immunotherapy. Here, Huseni et al. identify IL-6 as a correlate of poor clinical response to atezolizumab (anti-PD-L1) therapy and demonstrate that IL-6 impairs anti-PD-L1 efficacy by restricting the anti-tumor functions of cytotoxic T cells.

## Introduction

Immune checkpoint inhibitors (ICIs) are approved therapies for multiple forms of cancer, but they fail to elicit durable clinical responses in the majority of patients.^1^ Identifying therapeutically actionable mechanisms of ICI resistance is therefore critical to maximize the benefit of cancer immunotherapy.

Despite the well-described roles of many cytokines in tumor development, they are not widely exploited as therapeutic targets in oncology. The pleiotropic cytokine interleukin 6 (IL-6) is associated with tumor progression and is thought to influence anti-tumor immunity through various mechanisms.^2^^,^^3^^,^^4^ Interestingly, plasma IL-6 was associated prognostically with reduced survival of ICI-treated melanoma patients.^5^^,^^6^ Recent pre-clinical studies have also implicated IL-6 as a potential driver of ICI resistance,^5^^,^^7^^,^^8^^,^^9^^,^^10^ but the underlying mechanisms remain unclear.

In this study, we identified high IL-6 levels as a feature of atezolizumab (anti-PD-L1)-resistant disease in patients with advanced cancers. IL-6 restrained effector differentiation of CD8^+^ T cells (also known as cytotoxic T lymphocytes, or CTLs), and high plasma IL-6 correlated with reduced effector gene expression in CTLs from cancer patients. In pre-clinical tumor models, IL6R blockade or genetic ablation of CTL-intrinsic IL-6 signaling synergized with anti-PD-L1 therapy to enhance anti-tumor CTL responses, leading to improved tumor control.

## Results

### IL-6 is associated with poor clinical activity of atezolizumab (anti-PD-L1)

**Figure 1:**
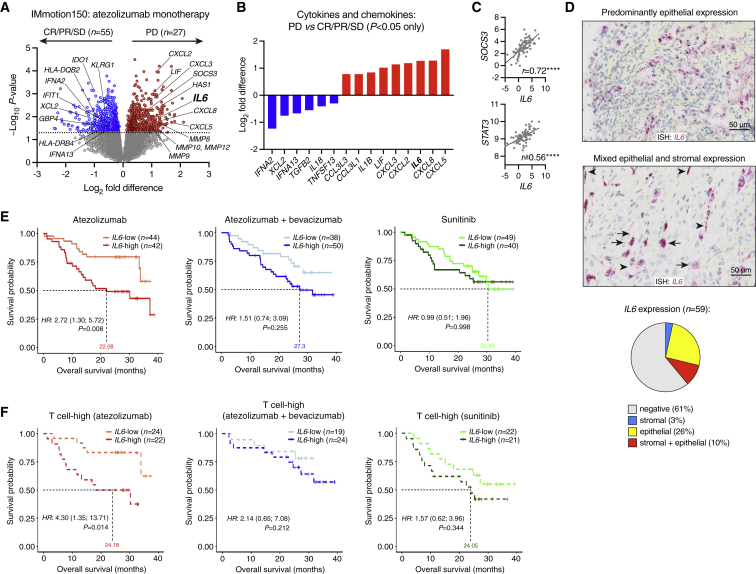
*IL6* expression associates with poor clinical outcome in atezolizumab-treated patients with metastatic RCC (A–C) RNA-seq analysis of pre-treatment tumor samples from the atezolizumab monotherapy arm of IMmotion150. (A) Differential gene expression analysis (Limma-voom), comparing PD (progressive disease) versus SD (stable disease), PR (partial response), or CR (complete response). Nominal p values are shown. (B) Differentially expressed cytokine and chemokine genes. (C) Pearson correlation of *IL6* with *SOCS3* and *STAT3*. ∗∗∗∗p < 0.0001. (D) ISH analysis of *IL6* mRNA in RCC tumors from IMmotion150 (n = 59). Black arrows, epithelial cell expression; arrowheads, stromal cell expression. Scale bar, 50 μm. Pie chart: proportions of tumors with *IL6* expression (staining in ≥1% of cells) in epithelial cells only (yellow), stromal cells only (blue), or both epithelial and stromal cells (red). (E) Association of tumor *IL6* mRNA with overall survival (OS) in IMmotion150. (F) Association of tumor *IL6* mRNA with OS in patients with high intratumoral T cell signature expression (>median). In (E) and (F), HR (+/− 95% CI) and p values were adjusted after multivariate analysis including the following co-variates: MSKCC (Memorial Sloan Kettering Cancer Center) prognostic risk score, previous nephrectomy, and liver metastasis.

To identify potential drivers of anti-PD-L1 resistance, we evaluated gene expression by RNA-seq in pre-treatment tumor biopsies from IMmotion150, a randomized phase II trial of previously untreated metastatic renal cell carcinoma (RCC) comparing atezolizumab with or without bevacizumab versus the tyrosine kinase inhibitor sunitinib.^11^ Clinical cohorts evaluated in this study are described in Figure S1A and Tables S1–S3. Among patients treated with atezolizumab monotherapy, differential expression analysis comparing those who developed progressive disease (PD) versus those with evidence of disease control revealed various inflammatory factors associated with PD, including the cytokine *IL6* (Figure 1A). Among other cytokines and chemokines associated with PD, only *CXCL5* and *CXCL8* were more highly ranked than *IL6* (Figure 1B). Ingenuity Pathway Analysis identified IL-6 as a putative upstream regulator of PD-associated genes (p = 3.16e−9), in addition to the IL-6 receptor (IL6R; p = 1.04e−11) and the IL-6-induced transcription factor STAT3 (signal transducer and activator of transcription 3; p = 1.45e−9). *IL6* correlated strongly with *STAT3* and the STAT3-response gene *SOCS3*, consistent with active IL-6 signaling (Figure 1C). Tumor *IL6* mRNA (evaluated in n = 59 evaluable cases of RCC using *in situ* hybridization [ISH]) was observed mainly in epithelial and stromal cells (Figure 1D).

To confirm the association of IL-6 with poor atezolizumab efficacy, we next evaluated long-term patient survival. After multivariate correction, high tumor *IL6* (>median) was associated significantly with poor overall survival (OS) only in patients who received atezolizumab monotherapy (hazard ratio [HR]: 2.72, 95% confidence interval [CI]: 1.30, 5.72, p = 0.008; Figure 1E), including those with high expression of a CD8^+^ T cell signature (HR: 4.30, 95% CI: 1.35, 13.71*,* p = 0.014; Figure 1F).^11^^,^^12^

IL-6 protein was significantly higher in pre-treatment plasma samples from RCC (IMmotion150) patients compared with healthy controls (Figure S1B) and correlated significantly with tumor *IL6* mRNA (*r* = 0.32, p < 0.0001; Figure S1C). High plasma IL-6 (>10 pg/mL; Figures S1D and S1E) was associated with reduced OS in all treatment arms (Figure S1F). Similarly, in atezolizumab-treated patients with metastatic triple-negative breast cancer (TNBC) from the PCD4989g clinical trial,^13^ or with metastatic urothelial bladder carcinoma (UC) from the IMvigor210 and IMvigor211 trials,^14^^,^^15^^,^^16^ plasma IL-6 was elevated compared with healthy controls (Figure S1B) and associated with poor OS in multivariate survival analysis (Figures S1G–S1I). The IL-6-driven acute phase response protein CRP (C-reactive protein) was similarly associated with plasma IL-6 and poor OS in all three cancer types (Figures S1J–S1M).

### Dual blockade of IL6R and PD-L1 improves tumor control and CTL function

**Figure 2:**
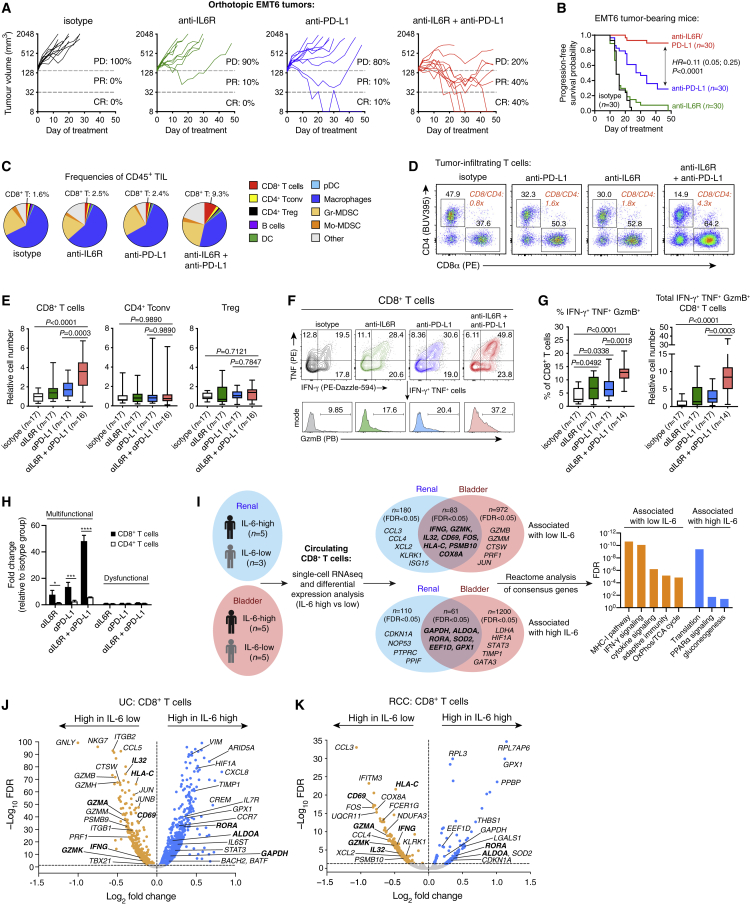
IL-6 inhibits anti-PD-L1 efficacy and anti-tumor CTL response (A–H) Treatment of EMT6 tumor-bearing mice with antibodies targeting PD-L1 and/or IL6R. (A) Individual tumor growth curves (n = 10 per group) from one of three independent studies. PD, progressive disease; PR, partial response; CR, complete response. (B) Progression-free survival (time to 5x increase in tumor volume) of mice pooled from three independent studies, analyzed using log rank test. (C) Cellular composition of CD45^+^ tumor-infiltrating leukocytes, from one of three experiments. (D) Balance of CD4^+^ and CD8^+^ T cells (CTLs) among total TCRβ^+^ tumor-infiltrating T cells. Data are concatenated from n = 5 mice per group, from one of three studies. (E) Relative abundance (normalized to tumor weight) of CTLs, conventional CD4^+^ T cells, and regulatory (CD4^+^ Foxp3^+^) T cells in EMT6 tumors, pooled from three experiments. Groups compared using one-way ANOVA with Holm-Sidak’s multiple comparisons test. (F) Effector phenotype of tumor-infiltrating CTLs after re-stimulation with PMA/ionomycin. (G) Frequencies of polyfunctional cells among tumor-infiltrating CTLs (left panel), and their absolute abundance relative to isotype control (right panel). Data pooled from three experiments. Groups compared using one-way ANOVA with Holm-Sidak’s multiple comparisons test. (H) Relative abundance (mean ± SEM) of polyfunctional CTLs and IFN-γ^+^TNF^+^ CD4^+^ T cells (multifunctional cells) vs. dysfunctional cells (IFN-γ^−^ TNF^−^ GzmB^−^ CD8^+^ T cells, or IFN-γ^−^ TNF^−^ CD4^+^ T cells), from one of three experiments with n = 3–5 per group. Groups compared using one-way ANOVA with Holm-Sidak’s multiple comparisons test. ∗p = 0.0143, ∗∗∗p = 0.0001, ∗∗∗∗p < 0.0001. (I–K) Single-cell RNA-seq analysis of pre-treatment peripheral blood CD8^+^ T cells from patients with RCC (from IMmotion150) or UC (from IMvigor210). Differential expression analysis was performed on CD8^+^ T cells from patients with high (>10 pg/mL) versus low plasma IL-6 (separate analyses for each cancer type). Genes identified as concordant in both cohorts (“consensus genes”) were evaluated for Reactome pathway enrichment (I). Volcano plots of differential gene expression are shown for UC and RCC samples in (J) and (K), respectively.

To determine if IL-6 affects anti-PD-L1 activity, we first examined the syngeneic EMT6 mouse model of TNBC (Figure S2A). EMT6 proliferation *in vitro* was unaffected by IL-6 (Figure S2B). Compared with either anti-IL6R or anti-PD-L1 monotherapy, combined blockade of IL6R and PD-L1 significantly improved tumor control and progression-free survival (HR = 0.11, p < 0.0001; Figures 2A and 2B). Analysis of fluorescence-activated cell sorting (FACS)-purified tumor populations (EMT6 cells, T cells, myeloid cells, and fibroblasts) identified inflammatory Ly6C^+^ cancer-associated fibroblasts as the main IL-6 producers (Figures S2C–S2D), consistent with prior studies.^17^^,^^18^^,^^19^

We next characterized tumor-infiltrating leukocytes at a time point prior to tumor regression (day 11). Combination therapy increased CTL frequency and abundance compared with anti-PD-L1 or anti-IL6R monotherapy, but it had little effect on regulatory T cells (Tregs), conventional CD4^+^ T cells, or myeloid cells (Figures 2C–2E, S2E), resulting in a significantly increased CD8^+^ to CD4^+^ T cell ratio (Figure 2D). Combination therapy also increased the frequency and abundance of polyfunctional (IFN-γ^+^TNF^+^GzmB^+^) CTLs (Figures 2F and 2G), with a comparatively modest effect on IFN-γ^+^TNF^+^ (Th1) CD4^+^ T cells (Figure 2H). Dysfunctional (IFN-γ^−^TNF^−^GzmB^−^) CTL abundance was unaffected (Figure 2H). While IL-6 can promote development of IL-17-producing CD4^+^ (Th17) and CD8^+^ (Tc17) T cells,^2^ these were rare in tumors and draining lymph nodes, and their abundance was unaffected by any treatment (Figures S2F–S2H). We next evaluated two additional tumor models: CT26 (anti-PD-L1-resistant) and MC38 (partially anti-PD-L1-responsive). Consistent with EMT6, combination therapy in both models improved tumor control and enhanced anti-tumor CTL responses compared with anti-PD-L1 alone (Figures S2I–S2O).

### High IL-6 is associated with impaired CTL function in cancer patients

To explore the relationship between IL-6 and CTL in cancer patients, we selected patients from the IMmotion150 (RCC) and IMvigor210 (UC) trials with high (>10 pg/mL) or low plasma IL-6 and performed 10x single-cell RNA sequencing of their pre-treatment peripheral blood mononuclear cells. CTLs were identified from n = 8 RCC patients (n = 5 IL-6-high and n = 3 IL-6-low) and n = 10 UC patients (n = 5 IL-6-high and n = 5 IL-6-low; Figures S3A and S3B). Differential expression analysis comparing CTLs from IL-6-high versus IL-6-low patients identified numerous genes that were shared between the RCC and UC datasets (Figures 2I–2K). Concordant genes associated with high IL-6 were related to glucose metabolism and protein synthesis pathways, the latter driven largely by ribosomal genes, suggesting a transcriptionally quiescent state (Figure 2I). In contrast, CTLs from IL-6-low patients showed high expression of genes associated with activation and effector function (including *CD69*, *IL32, IFNG*, and *GZMK*), as well as enrichment of IFN signaling, the MHC-I pathway, and oxidative phosphorylation (OxPhos) (Figure 2I, Table S4).

Higher resolution clustering revealed several CTL subpopulations that associated strongly with plasma IL-6 status (Figures S3C–S3H). Clusters of polyfunctional effector cells expressing *IFNG*, *GZMB*, and various chemokines were identified in both the RCC and UC datasets (C5 and C2, respectively), and they occurred almost exclusively in IL-6-low patients. An additional terminal effector-like population was associated with low IL-6 in UC patients (C0), while IL-6-low RCC patients harbored cells with high expression of *GZMA*, *GZMK*, and *TCF7*, suggesting a state of early activation (C4). In contrast, the majority of cells from IL-6-high patients appeared to be inactive or functionally impaired, with low expression of *IFNG* and *GZMB* (e.g., UC cluster C4; Figures S3C–S3H).

### IL-6 inhibits CTL effector differentiation

**Figure 3:**
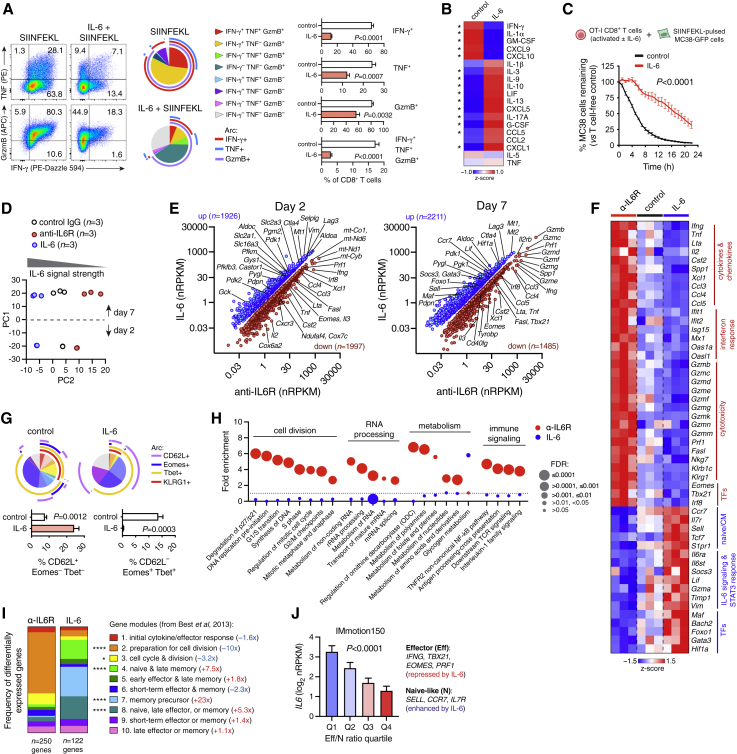
IL-6 blocks CTL effector differentiation (A) Splenocytes from OT-I mice were stimulated with SIINFEKL peptide +/− IL-6 and analyzed by flow cytometry on day 7. Boolean analysis of IFN-γ, TNF, and GzmB expression in CTLs is shown. Groups (mean +/− SEM of n = 4 technical replicates) were compared by t test and represent one of three independent experiments. (B) Cytokine secretion (measured by Luminex multiplex assay) by FACS-purified CTLs activated with anti-CD3/CD28 +/− IL-6 for 3 days ∗p < 0.05 (t test; n = 3 technical replicates per condition). (C) OT-I CTLs were activated as described in (A) and co-cultured with SIINFEKL-pulsed MC38.GFP cells at a 5:1 effector/target ratio. MC38 destruction was quantified using Incucyte live-cell imaging. Groups compared using two-way ANOVA (n = 4 technical replicates per condition); data indicate mean ± SEM, and are representative of three independent experiments. (D–I) OT-I splenocytes activated with SIINFEKL peptide in the presence of IL-6, control IgG, or anti-IL6R antibody. CTLs were FACS-purified for RNA-seq analysis after 2 and 7 days (n = 3 technical replicates per condition/time point). Principal components analysis (PCA) is shown in (D). (E) Differentially expressed genes (FDR <0.05 and absolute fold change >1.5). (F) Heatmap of representative differentially expressed genes (day 7). (G) Boolean flow cytometry analysis of OT-I CTLs at day 7. Groups (mean +/− SEM of n = 4 technical replicates) compared using *t* tests, from one of three independent experiments. (H) Reactome pathway analysis of differentially expressed genes between IL-6- and anti-IL6R-treated cells at day 7. (I) Distribution of differentially expressed genes (day 7) among differentiation modules from Best et al.^25^ ∗p = 0.0239, ∗∗∗∗p < 0.0001 (Fisher’s exact test). Fold differences refer to IL-6 versus anti-IL6R treatment. (J) Tumor RNA-seq analysis from IMmotion150. Gene modules (average *Z* scores) associated with effector or naive-like CTLs were used to calculate an effector/naive-like (Eff/N) ratio. *IL6* expression across Eff/N quartiles (mean +/− SEM, n = 65–66 per group) was compared using one-way ANOVA.

We next performed a series of *in vitro* studies to explore the mechanistic basis of reduced CTL function under IL-6-high conditions. IL-6 can influence CTL metabolism and promote unconventional differentiation states (Tc17, Tc21, Tc22) under specific conditions,^20^^,^^21^^,^^22^^,^^23^^,^^24^ but its impact on classical effector differentiation is not well understood. Consistent with our findings *in vivo*, IL-6 inhibited expression of IFN-γ, TNF, and GzmB by CTL from OT-I TCR-transgenic mice after activation with SIINFEKL peptide (Figure 3A). IL-6 also reduced the rate of cell division, but this effect was offset by improved cell viability (Figure S4A). We confirmed these findings by activating splenic CTLs from wild-type (WT) or IL6R.ko mice with anti-CD3/CD28 antibodies in the presence or absence of IL-6 (classical signaling) or hyper-IL-6 (a complex of IL-6 and soluble IL6R that activates *trans* signaling via direct engagement of gp130).^3^ As expected, IL6R.ko T cells were unaffected by IL-6 but remained susceptible to functional impairment by hyper-IL-6 (Figures S4B and S4C). IL-6 inhibited secretion of IFN-γ, GM-CSF, and IFN-responsive chemokines (CXCL9 and CXCL10), while it induced production of IL-10 and factors related to Th2/Th17 responses (IL-13 and IL-17A; Figure 3B). IL-6 repressed effector functions of both naive and memory CTLs (Figure S4D), and it had a similar effect on naive human CTLs (Figure S4E). Importantly, IL-6-conditioned OT-I CTLs were attenuated in their ability to kill SIINFEKL-loaded MC38 cells (Figure 3C).

While IL-6 inhibited CTL activation *in vitro* (Figure 3), IL6R blockade *in vivo* showed clear effects only in combination with anti-PD-L1 (Figure 2). Optimized T cell activation *in vitro* is likely more efficient than activation *in vivo*, which could influence the impact of IL-6. Indeed, PD-1 blockade *in vivo* was recently shown to enhance TCR signal strength in CD4^+^ T cells.^26^ To explore this concept further, we identified a sub-optimal SIINFEKL concentration (1 ng/mL) at which PD-L1 blockade enhanced OT-I CTL activation (Figure S4F). IL-6 had little effect on T cells activated with 1 ng/mL peptide alone but prevented expansion of IFN-γ^+^ CTLs driven by anti-PD-L1 (Figures S4G and S4H). Thus, the inhibitory effect of IL-6 is most apparent when T cells are activated by strong TCR/co-stimulatory signals.

### IL-6 promotes naive/memory-like features in CTLs

To further characterize the effects of IL-6, we activated CTLs in the presence of IL-6 or anti-IL6R antibody (to block endogenous signaling) and analyzed them by RNA-seq after 2 and 7 days (Figures 3D–3F). IL-6 suppressed expression of effector genes (e.g., *Ifng*, *Gzmb*, *Prf1*, *Klrg1*), chemokines, and transcription factors necessary for effector differentiation including *Tbx21* and *Eomes* (Figures 3E and 3F).^27^^,^^28^^,^^29^ Conversely, IL-6 promoted expression of transcription factors that oppose effector differentiation, including *Foxo1* and *Bach2*,^30^^,^^31^ and genes associated with naive or central memory (CM) cells such as *Sell*, *Ccr7,* and *Il7r* (Figures 3E and 3F). We confirmed by flow cytometry that IL-6 suppressed expression of Tbet, Eomes, and KLRG1, but it promoted retention of CD62L (Figure 3G). Reactome pathway analysis suggested that IL-6 suppressed processes related to immune signaling, cell division, metabolism, and RNA processing (Figure 3H, Table S5). Mapping IL-6-regulated genes onto transcriptional modules associated with different stages of CTL differentiation (Best et al.)^25^ confirmed that IL-6 suppressed genes related to CTL activation and cell cycle engagement (modules 2/3), while it promoted genes related to naive, memory precursor, and late memory states (modules 4/7/8; Figure 3I). We next used tumor RNA-seq data from IMmotion150 to evaluate gene expression associated with effector (Eff) versus naive-like (N) CTLs. Intriguingly, *IL6* was associated inversely with the Eff/N expression ratio (p < 0.0001; Figure 3J).

### IL-6 controls expression of diverse T cell regulators

To address whether IL-6 effects are reversible, we activated CTLs and stimulated them with IL-6 or hyper-IL-6 for the first 2 days only, or during days 3–7. IL-6/hyper-IL-6 signaling on days 0–2 attenuated effector function at day 7, whereas exposure after day 2 had no effect (Figure S4I). CTL downregulated *Il6r* expression (and to a lesser extent *Il6st*/gp130) after TCR stimulation, suggesting that T cells may lose sensitivity to IL-6 after priming (Figure S4J). However, primed cells remained capable of activating STAT3 in response to IL-6 or hyper-IL-6 (Figure S4K). Similarly, effector memory CTLs displayed lower IL6R and gp130 levels than naive or CM cells (Figure S4L) but remained capable of activating STAT3 in response to IL-6 (Figure S4M).

**Figure 4:**
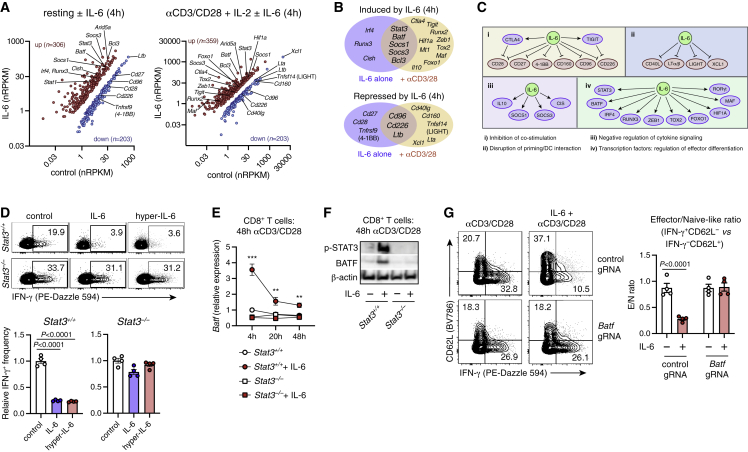
IL-6 regulates CTLs via STAT3-dependent BATF induction (A–C) RNA-seq analysis of WT naive CTLs stimulated with IL-6 +/− anti-CD3/CD28 antibodies for 4 h. (A) Differentially expressed genes (FDR <0.05 and absolute fold change >2). (B) IL-6-regulated genes with potential roles in CTL differentiation, and their functional categorization (C). (D) IFN-γ expression in WT or STAT3.ko CTLs (from CD4-Cre x *Il6r*^*loxP*^^*/loxP*^ mice) activated with anti-CD3/CD28 +/− IL-6 or hyper-IL-6 for 3 days. Groups (mean ± SEM of n = 4 technical replicates) compared by t test; data representative of three independent experiments. (E) *Batf* mRNA expression (qRT-PCR) in WT or STAT3.ko CTLs activated +/− IL-6. ∗∗p < 0.01, ∗∗∗p < 0.001 (WT vs. STAT3.ko; t tests). Data points indicate mean +/− SEM of n = 4 technical replicates, from one of two independent experiments. (F) Western blot of BATF and p-STAT3 (Y705) in activated WT or STAT3.ko CTLs. Data represent one of two independent experiments. (G) BATF CRISPR-ko or control CTLs were activated +/− IL-6 and analyzed on day 3 (groups compared by t test; mean ± SEM of n = 4 technical replicates). Data representative of two independent experiments.

To determine if IL-6 pre-conditioning affects CTL differentiation upon subsequent TCR stimulation, we treated CTLs with IL-6 for 1 day; then we withdrew IL-6 and stimulated with anti-CD3/CD28. IL-6 pre-treatment suppressed effector function at day 3 (Figure S4N), suggesting that IL-6 alters expression of downstream regulators that provide durable control of T cell function, regardless of ongoing IL-6 signaling. To identify candidate regulators, we performed RNA-seq analysis of naive CTLs stimulated with IL-6 alone, or with IL-6 and anti-CD3/CD28 for 4 h (Figure 4A). Intriguingly, *Ifng* was induced by IL-6 at 4 h, confirming that proximal IL-6 signaling does not block effector function directly (Figure S4O). IL-6 suppressed expression of numerous genes with known importance for CTL activation, including co-stimulatory receptors (*Cd28*, *Cd226*) and mediators of dendritic cell cross-talk (*Cd40lg*, *Xcl1*), while it induced expression of co-inhibitory receptors (*Ctla4*, *Tigit*), repressors of inflammatory signaling (*Il10* and SOCS-family members), and diverse transcription factors (*Batf*, *Foxo1*, *Hif1a*, *Tox2*; Figures 4B and 4C).

To identify mediators of IL-6-driven CTL dysfunction, we first confirmed a requirement for STAT3, the primary transcription factor activated by IL-6.^3^ As expected, IFN-γ production by STAT3-deficient CTLs was unaffected by IL-6 (Figure 4D). Among additional IL-6-regulated factors (Figures 4A–4C), we were intrigued by BATF (basic leucine zipper transcription factor, ATF-like), which restricts expression of CTL effector genes (including IFN-γ and perforin)^32^ and can be induced by IL-6.^23^^,^^33^ Indeed, IL-6 triggered rapid STAT3-dependent BATF expression in CTLs (Figures 4E and 4F), and knockout of *Batf* using CRISPR-Cas9 abrogated the effect of IL-6 on CTL effector differentiation (Figures 4G and S4P). Thus, IL-6 may impair CTL function through STAT3-dependent induction of BATF.

### CTL-intrinsic IL-6 signaling inhibits anti-PD-L1 efficacy

To evaluate the impact of CTL-intrinsic IL-6 signaling *in vivo*, we adoptively transferred naive CTLs from CD90.1^+^ OT-I mice that were *Il6r*^*wt/wt*^ or *Il6r*^*−/−*^ to WT CD90.2^+^ recipients bearing ovalbumin-expressing EG.7 tumors. Combined with ovalbumin immunization (Figures S5A and S5B), PD-L1 blockade increased the polyfunctional anti-tumor CTL response in mice that received *Il6r*^*−/−*^ OT-I cells, but not *Il6r*^*wt/wt*^ cells (Figures S5C and S5D).

**Figure 5:**
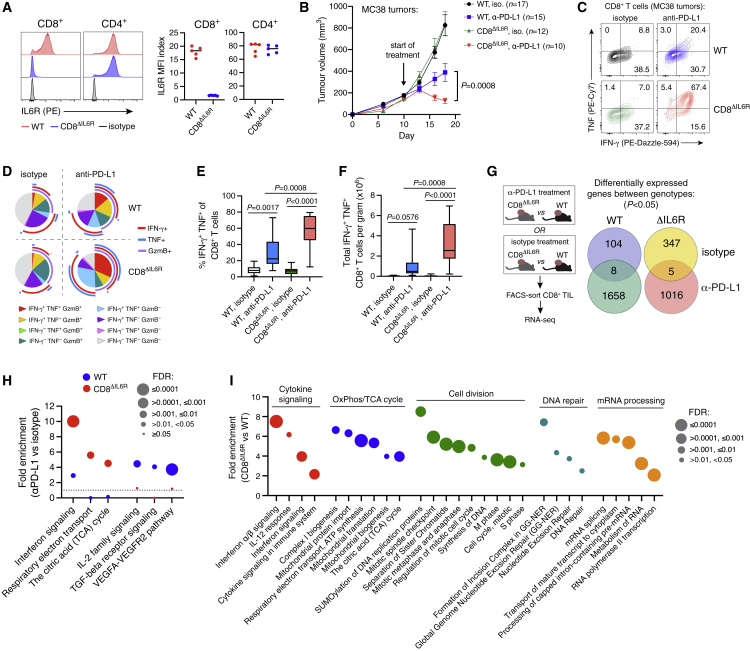
CTL-intrinsic IL-6 signaling impairs anti-PD-L1 efficacy (A) IL6R expression on lymph node T cells from CD8^ΔIL6R^ mice or WT littermate controls (n = 5 mice per group). (B) Tumor growth (mean +/− SEM) in CD8^ΔIL6R^ mice and WT littermates treated with anti-PD-L1 or control antibodies, pooled from two independent studies and compared using two-way ANOVA. (C–F) Cytokine expression in tumor-infiltrating CTLs after 1 week of treatment. Representative staining and Boolean analysis are shown in (C) and (D), respectively. Frequencies of IFN-γ^+^ TNF^+^ cells among tumor-infiltrating CTLs (E), and their absolute number (F), compared using one-way ANOVA with Holm-Sidak’s multiple comparisons test (n = 9–12 mice per group). Data pooled from two independent studies. (G–I) RNA-seq analysis of FACS-purified tumor-infiltrating CTLs from CD8^ΔIL6R^ mice or WT littermates. Mice with established MC38 tumors (∼150 mm^3^) were treated with anti-PD-L1 or control antibodies for 7 days (n = 4–5 mice per group). (G) Differentially expressed genes between WT and CD8^ΔIL6R^ CTLs. Separate comparisons were made based on treatment. (H) Reactome pathway analysis of anti-PD-L1-driven protein-coding genes (p < 0.05) in WT and CD8^ΔIL6R^ CTLs. (I) Reactome pathway analysis of the top 500 protein-coding genes (ranked by p value) that were significantly associated with CD8^ΔIL6R^ CTLs during anti-PD-L1 treatment.

We next crossed *E8i.Cd8a.Cre* and *Il6r*.*loxP* mice to generate animals with CTL-restricted IL6R deficiency (CD8^ΔIL6R^; Figures 5A and S5E). CTLs from CD8^ΔIL6R^ mice were unresponsive to classical IL-6 signaling, whereas CD4^+^ T cells showed normal IL-6 responsiveness (Figures S5F and S5G). MC38 tumors grew slowly in CD8^ΔIL6R^ mice (Figure S5H); thus, only CD8^ΔIL6R^ mice with relatively large tumors (comparable in size to those of WT mice) were selected for therapeutic studies. Compared with WT littermates, CD8^ΔIL6R^ mice showed greater tumor control (Figure 5B) and stronger induction of anti-tumor CTL responses during PD-L1 blockade (Figures 5C–5F and S5I). This phenotype was not due to obvious baseline differences in T cell development or function (Figure S5J).

RNA-seq analysis of intratumoral CD8^ΔIL6R^ versus WT CTLs revealed substantial differences in gene expression (Figure 5G). Anti-PD-L1 treatment induced genes related to IL-2, TGFβ, and VEGF signaling in WT CTLs, whereas it drove genes associated with IFN response and OxPhos in IL6R-deficient CTLs (Figure 5H, Table S5). We next evaluated genotype-dependent expression in CTLs from mice treated with anti-PD-L1. No differences were observed in co-inhibitory molecule expression (Figure S5K). IL6R-deficient CTLs expressed high amounts of genes related to IFN response, OxPhos, mitochondrial biogenesis, cell cycle, DNA repair, and mRNA processing (Figure 5I) and low amounts of naive-associated factors (Figure S5L). Genes associated with CD8^ΔIL6R^ CTL were found predominantly in modules 2 and 3 (early activation and cell division) from Best et al.,^25^ whereas WT-associated genes were related to modules 7 and 8 (naive and memory precursor; Figure S5M). Overall, these findings suggest that during PD-L1 blockade, IL-6 acts on CTLs to restrain their effector differentiation and impair therapeutic efficacy.

## Discussion

Our clinical observations corroborate the recently described prognostic association between plasma IL-6/CRP and poor survival of ICI-treated melanoma patients,^6^^,^^34^ and they identify high tumor *IL6* expression as a predictor of poor atezolizumab monotherapy outcome in RCC. This relationship may be causal based on our experimental data, which demonstrate that IL-6 can impair anti-PD-L1 efficacy via direct functional inhibition of CTLs. Consistent with the association between plasma IL-6 and CTL dysfunction in patients with COVID-19,^35^^,^^36^^,^^37^^,^^38^ we identified polyfunctional effector CTLs almost exclusively in cancer patients with low plasma IL-6.

IL-6 was reported recently to also inhibit anti-CTLA4 efficacy in mouse tumor models.^39^ Notably, while IL-6 blockade promoted anti-tumor immunity in combination with anti-CTLA4, it ameliorated pathology driven by autoreactive Th17 responses,^39^ suggesting that IL-6/IL6R blockade may have the added benefit of attenuating immune-related adverse events.

IL-6 can potentially influence anti-PD-L1 efficacy through several mechanisms, including support of cancer cell fitness,^2^^,^^4^ inhibition of Th1 responses,^5^^,^^8^^,^^10^ promotion of immunosuppressive myeloid cells,^7^^,^^9^^,^^40^^,^^41^^,^^42^ or disruption of conventional dendritic cells (cDCs).^43^ In our studies, IL6R blockade had little impact on myeloid populations, and anti-IL6R/PD-L1 combination therapy did not affect Th1 responses reproducibly. In contrast, combination therapy enhanced CTL responses in several tumor models, consistent with the inhibitory effect of IL-6 on CTLs *in vitro*. While this could be related to impaired cDC1s,^43^ we observed only modest changes in cDCs during combination therapy, and CTL-restricted IL6R deficiency enhanced anti-PD-L1 efficacy. Thus, although IL-6 can potentially influence several cell types to impair ICI activity, direct effects on CTLs may also be important for this process.

IL-6-mediated CTL inhibition required STAT3-dependent expression of BATF, consistent with prior observations implicating STAT3 and BATF as regulators of CTL function.^22^^,^^32^^,^^44^^,^^45^^,^^46^ However, the complete mechanism *in vivo* is likely complex and context dependent, since IL-6 controls expression of numerous T cell regulators, including the immunotherapy targets CTLA4 and TIGIT. IL-6 also promoted expression of factors necessary for memory formation, including Foxo1, IL-10, and Bach2,^30^^,^^31^^,^^44^^,^^47^^,^^48^^,^^49^ consistent with its role in promoting CD4^+^ T cell memory.^50^ Although CTLs with progenitor-like properties are important for sustained anti-tumor immunity,^51^ successful immunotherapy requires their differentiation into cytotoxic effector cells.^52^^,^^53^^,^^54^^,^^55^ Thus, by promoting progenitor/memory-like properties, IL-6 may support the longevity of anti-tumor CTLs but nevertheless limit ICI efficacy by impairing their development into effector cells.

Direct inhibition of CTLs by IL-6 is mechanistically distinct from other cytokine-dependent processes that can potentially inhibit ICI efficacy, including recruitment of inhibitory myeloid cells by VEGF,^56^ IL-1β,^57^ IL-8,^58^^,^^59^ or LIF,^60^ suppression of tumor CTL infiltration by TGFβ,^61^ and support of Treg function by IL-23.^62^^,^^63^ Importantly, therapies targeting IL-6 and IL6R are approved for several conditions including rheumatoid arthritis, cytokine release syndrome, giant cell arteritis, and multicentric Castleman disease.^64^ Given the extensive clinical experience with IL-6 inhibitors, and the pressing need to improve upon existing immunotherapies, combination of ICIs with approved anti-IL-6/IL6R agents warrants investigation in cancer patients.

### Limitations of the study

Interpretation of our clinical data is limited by their retrospective nature, and prospective studies are necessary to fully evaluate the association of IL-6 with immunotherapy efficacy. IL-6 can potentially influence anti-PD-L1 response through many cell types, and our study does not provide a comprehensive assessment of its cell-intrinsic effects in non-CTLs. Further experiments with lineage-specific deletion of IL6R, combined with analyses such as single-cell RNA-seq, could provide additional mechanistic information. Finally, clinical studies of IL-6 pathway inhibitors in combination with ICIs will be necessary to fully evaluate the therapeutic translatability of our findings.

## STAR★Methods

### Key resources table

**Table:** 

REAGENT or RESOURCE	SOURCE	IDENTIFIER
**Antibodies**		

Anti-PD-L1 (mouse IgG2A, clone 6E11)	Genentech	N/A
Anti-IL6R (mouse IgG2A, clone MR16-1)	Genentech	N/A
Mouse IgG2A isotype control (anti-gp120)	Genentech	N/A
Anti-mouse-DEC205-ovalbumin	Genentech	N/A
Anti-mouse-CD40 (clone FGK4.5)	Genentech	N/A
Rabbit anti-phospho-STAT3 (Y705; clone D3A7)	Cell Signaling Technology	Cat#9145; RRID:AB_2491009
Rabbit anti-STAT3 (clone D1B2J)	Cell Signaling Technology	Cat#30835; RRID:AB_2798995
Mouse anti-β-actin-HRP (clone AC-15)	Sigma-Aldrich	Cat#A3854; RRID:AB_262011
Rabbit anti-BATF (clone D7C5)	Cell Signaling Technology	Cat#8638; RRID:AB_11141425
Hamster anti-mouse CD3e (clone 145-2C11)	BD Biosciences	Cat#550275; RRID:AB_393572
Hamster anti-mouse CD28 (clone 37.51)	BD Biosciences	Cat#553295; RRID:AB_394764
Rat anti-mouse CD16/32 (clone 2.4G2)	BD Biosciences	Cat#553142; RRID:AB_394657
Anti-mouse CD45-BV510 (clone 30-F11)	BD Biosciences	Cat#563891; RRID:AB_2734134
Anti-mouse Thy1.2-efluor450 (clone 53-2.1)	eBioscience	Cat#48-0902-82; RRID:AB_1272200
Anti-mouse Thy1.2-alexafluor700 (clone 53-2.1)	Biolegend	Cat#140324; RRID:AB_2566740
Anti-mouse Thy1.1-alexafluor488 (clone OX-7)	Biolegend	Cat#202506; RRID:AB_492882
Anti-mouse CD3ε-PE/Cy5.5 (clone 145-2C11)	Biolegend	Cat#100310; RRID:AB_312675
Anti-mouse CD4-BUV395 (clone GK1.5)	BD Biosciences	Cat#563790; RRID:AB_2738426
Anti-mouse CD4-BV785 (clone GK1.5)	Biolegend	Cat#100453; RRID:AB_2565843
Anti-mouse CD8a-BB515 (clone 53-6.7)	BD Biosciences	Cat#564422; RRID:AB_2738801
Anti-mouse CD8a-PE (clone 53-6.7)	Biolegend	Cat#100708; RRID:AB_312747
Anti-mouse CD8a-BUV737 (clone 53-6.7)	BD Biosciences	Cat#612759; RRID:AB_2870090
Anti-mouse CD11b-alexafluor700 (clone M1/70)	Biolegend	Cat#101222; RRID:AB_493705
Anti-mouse Gr1-PE-Cy5.5 (clone RB6-8C5)	eBioscience	Cat#35-5931-82; RRID:AB_469740
Anti-mouse CD11c-PE/Dazzle594 (clone N418)	Biolegend	Cat#117348; RRID:AB_2563655
Anti-mouse MHCII (I-A/I-E)-FITC (clone M5/114.15.2)	eBioscience	Cat#11-5321-85; RRID:AB_465233
Anti-mouse CD64-PE/Cy7 (clone X54-5/7.1)	Biolegend	Cat#139314; RRID:AB_2563904
Anti-mouse CD169-PE/Cy7 (clone 3D6.112)	Biolegend	Cat#142412; RRID:AB_2563911
Anti-mouse B220-BUV737 (clone RA3-6B2)	BD Biosciences	Cat#612838; RRID:AB_2870160
Anti-mouse IL6R-PE (clone D7715A7)	Biolegend	Cat#115806; RRID:AB_313677
Anti-mouse gp130-PE (clone 4H1B35)	Biolegend	Cat#149403; RRID:AB_2566644
Anti-mouse Foxp3-efluor450 (clone FJK-16s)	eBioscience	Cat#48-5773-82; RRID:AB_1518812
Anti-mouse/human GzmB-Pacific blue (clone GB11)	Biolegend	Cat#515408; RRID:AB_2562196
Anti-mouse TNF-PE (clone MP6-XT22)	Biolegend	Cat#506306; RRID:AB_315427
Anti-mouse IFNγ-PE/Dazzle594 (clone XMG1.2)	Biolegend	Cat#505846; RRID:AB_2563980
Anti-mouse IL-17A-BV785 (clone C11-18H10)	Biolegend	Cat#506928; RRID:AB_2629787
Anti-human CD8a-BUV395 (clone RPA-T8)	BD Biosciences	Cat#563796
Anti-human TNF-alexafluor488 (clone MAb11)	Biolegend	Cat#502917; RRID:AB_493122
Anti-human IFNγ-APC (clone B27)	Biolegend	Cat#506510; RRID:AB_315443
Anti-human CD3 (clone OKT3)	eBioscience	Cat#16-0037-85; RRID:AB_468855
Anti-human CD28 (clone CD28.2)	BD Biosciences	Cat#555725; RRID:AB_396068

**Biological samples**		

Pre-treatment PBMC from Atezolizumab trials (IMmotion150 and IMvigor210)	Genentech	N/A
Healthy donor PBMC	Genentech	N/A
Renal cell cancer tissue blocks from IMmotion150	Genentech	N/A

**Chemicals, peptides, and recombinant proteins**		

TrueCut Cas9 Protein v2	Thermo Fisher Scientific	Cat#A36499
Recombinant mouse IL-6	R&D Systems	Cat#406-ML
Recombinant mouse hyper-IL-6	R&D Systems	Cat# 9038-SR-025/CF
Recombinant human IL-6	R&D Systems	Cat#206-IL
Recombinant human IL-2	R&D Systems	Cat#202-IL
SIINFEKL (OVA 257-264) peptide	AnaSpec	Cat#AS-60193-1
Fixable Viability Dye eFluor™ 780	Invitrogen	Cat#65-0865-14
Cell Stimulation Cocktail plus protein transport inhibitors (500X)	Invitrogen	Cat#00-4975-03
Brefeldin A (1000X)	eBioscience	Cat#00-4506-51

**Critical commercial assays**		

Foxp3/Transcription Factor Staining Buffer Set	eBioscience	Cat#00-5523-00
EasySep Mouse CD8^+^ T cell Isolation Kit	StemCell Technologies	Cat#19853
EasySep Mouse Naive CD8^+^ T cell Isolation Kit	StemCell Technologies	Cat#19858
EasySep Human Naive CD8^+^ T cell Isolation Kit	StemCell Technologies	Cat#19258
RNEasy Mini Kit	Qiagen	Cat#74104
iScript cDNA synthesis kit	Biorad	Cat#1708891

**Deposited data**		

Raw and analyzed bulk RNAseq data (mouse)	This paper	GEO: GSE199048
Tumor expression data (IMmotion150)	EGA	EGA: EGAS00001004387
Human PBMC single-cell RNAseq count data	EGA	EGA: EGAS00001004451, EGAS00001004458

**Experimental models: Cell lines**		

EMT6	Genentech	N/A
MC38	Genentech	N/A
MC38-GFP	Genentech	N/A
CT26	Genentech	N/A
EG7.OVA	Genentech	N/A

**Experimental models: Organisms/strains**		

Mouse: C57BL/6J	The Jackson Laboratory	JAX: 000664
Mouse: BALB/c	Charles River	Model# 028
Mouse: B6; SJL-*Il6ra*^*tm1.1Drew*^/J	The Jackson Laboratory	JAX: 012944
Mouse: Rosa26-Cre.ki (C57BL/6NTac-*Gt(ROSA)26Sor*^*tm16(cre)Arte*^)	Taconic	Model# 12524
Mouse: IL6R^−/−^ (B6; SJL-*Il6ra*^*tm1.1Drew*^/J x C57BL/6NTac-*Gt(ROSA)26Sor*^*tm16(cre)Arte*^	This paper	N/A
Mouse OT-I: C57BL/6^Tg(TcraTcrb)1100Mjb/J^	The Jackson Laboratory	JAX: 003831
Mouse: IL6R^−/−^ OT-1 (C57BL/6^Tg(TcraTcrb)1100Mjb/J^ x C57BL/6^*Il6r*−/−^)	This paper	N/A
Mouse: Cd4-Cre (B6.Cg-Tg(CD4-cre)1Cwi N9)	Taconic	Model# 4196
Mouse: Stat3^flox^ (B6.129S1-*Stat3*^*tm1Xyfu*^/J)	The Jackson Laboratory	JAX: 016923
Mouse: CD4^*ΔSTAT3*^ (B6.Cg-Tg(CD4-cre)1Cwi N9 x B6.129S1-*Stat3*^*tm1Xyfu*^/J)	This paper	N/A
Mouse: Cd8a-Cre (C57BL/6-Tg(Cd8a-cre)1Itan/J)	The Jackson Laboratory	JAX: 008766
Mouse: CD8^*ΔIL6R*^ (C57BL/6-Tg(Cd8a-cre)1Itan/J x B6; SJL-*Il6ra*^*tm1.1Drew*^/J)	This paper	N/A

**Oligonucleotides**		

Rpl19 (Mm02601633_g1)	Life Technologies	Cat#4331182
Il6ra (Mm01211445_m1)	Life Technologies	Cat#4331182
Il6st (Mm00439665_m1)	Life Technologies	Cat#4331182
Batf (Mm00479410_m1)	Life Technologies	Cat#4331182
Guide targeting exon 1 of Batf 5′ GGGGGTACCTGTTTGCCAG-3′	IDT	N/A
Alt-R CRISPR-Cas9 tracrRNA	IDT	Cat#1072534
Alt-R CRISPR-Cas9 negative control crRNA	IDT	Cat#1072544
IL6 in situ hybridization probe	Advanced Cell Diagnostics	Cat#2-1082

**Software and algorithms**		

FlowJo	FlowJo	https://www.flowjo.com/
Prism 9	GraphPad	https://www.graphpad.com/
R	The R Project for Statistical Computing	https://www.r-project.org/
Microsoft Excel for Mac	Microsoft	https://products.office.com/en-us/excel

### Resource availability

#### Lead contact

Further information and requests for resources and reagents should be directed to and will be fulfilled by the lead contact, Nathaniel West (west.nathaniel@gene.com).

#### Materials availability

This study did not generate new unique reagents.

### Experimental model and subject details

#### *In vivo* animal studies

C57BL/6J (JAX stock #000664), BALB/c (Charles River), C57BL/6.OT-I (JAX stock #003831),^65^ C57BL/6.Stat3^flox^ (JAX stock #016923),^66^ C57BL/6.CD4-cre (Taconic stock #4196),^67^ C57BL/6J.Il6ra^flox^ (JAX stock #012944),^68^ and C57BL/6.E8i.Cd8a-cre (JAX stock #008766)^69^ mice were bred and housed at Genentech under specific pathogen free (SPF) conditions. Whole-body IL6R.ko mice were generated by crossing C57BL/6J.Il6ra^flox^ and Rosa26-Cre.ki mice. Female mice were used for all studies and were 8–10 weeks old at the start of experiments. Experimental animals were housed at Genentech in standard rodent microisolator cages. Specific animal genotypes are indicated in figures or figure legends. For studies of transgenic mice, littermate controls were used where appropriate. All animal studies were approved by the Genentech Institutional Animal Care and Use Committee.

#### Human clinical studies

Patient demographics for the clinical trials analyzed in this study are provided in Tables S1, S2, and S3. All patients gave informed consent and studies were approved by their respective ethical review committees. For specific details of ethical review and study designs, see original publications for IMmotion150,^11^ PCD4989g,^13^ IMvigor210,^14^^,^^15^ and IMvigor211.^16^

#### Cell lines and primary cultures

The EMT6 murine mammary carcinoma (BALB/c), CT26 murine colon carcinoma (BALB/c), MC38 murine colon carcinoma (C57BL/6), and E.G7-OVA murine lymphoma (C57BL/6) cell lines were cultured in Roswell Park Memorial Institute (RPMI) 1640 medium plus 2 mM L-glutamine with 10% fetal bovine serum (FBS; Hyclone, Waltham, MA). Cell lines used in this study were negative for mycoplasma (based on Lonza Mycoalert and Stratagene Mycosensor assays) and authenticated by RNA-seq analysis. Sources of primary T cells used in *ex vivo* activation assays are specified in figure legends.

### Method details

#### Clinical sample collection

Plasma samples from TNBC patients were collected from PCD4989g (NCT01375842), a single-arm Phase I study that evaluated the clinical activity of atezolizumab in patients with locally advanced or metastatic malignancies, including TNBC. Bladder cancer plasma samples were collected in IMvigor210, a single-arm Phase 2 study investigating atezolizumab in UC patients (NCT02951767, NCT02108652) and in the Phase 3 UC trial IMvigor211 (NCT02302807) in which patients were treated with either chemotherapy (taxane or vinflunine) or atezolizumab as a second-line or higher treatment. RCC plasma and tumor samples were collected from IMmotion150 (NCT01984242), a phase II multicenter, randomized, open-label study investigating activity of atezolizumab and atezolizumab + bevacizumab versus sunitinib in metastatic clear cell renal carcinoma. Specimens from RCC patients were acquired <12 months before study treatment.

#### RNAseq profiling of tumor tissue

Whole-transcriptome profiles were generated using TruSeq RNA Access technology (Illumina). RNA-seq reads were first aligned to ribosomal RNA sequences to remove ribosomal reads. The remaining reads were aligned to the human reference genome (NCBI Build 38) using GSNAP version 2013-10-10 (Wu et al., 2016), allowing a maximum of two mismatches per 75 base sequence (parameters: ‘-M 2 -n 10 -B 2 -i 1 -N 1 -w 200000 -E 1-pairmax-rna = 200000 –clip-overlap). To quantify gene expression levels, the number of reads mapped to the exons of each RefSeq gene was calculated using the functionality provided by the R/Bioconductor package GenomicAlignments. The CD8^+^ T cell gene expression signature (GES) was defined in a previous publication for RCC.^11^

#### Plasma IL-6 assay

EDTA plasma samples were collected from patients and stored at −80°C. Plasma IL-6 was evaluated by previously qualified immunoassays on the multi-analyte platform Simple Plex Ella.^70^ The samples were diluted twofold in sample diluent and loaded onto the cartridge for data acquisition.

#### PBMC isolation and scRNAseq analysis

PBMC from UC and RCC patients were isolated using 50 mL Leucosep™ tubes (Greiner Bio-One International, Germany) and Ficoll-Paque™ PLUS (GE Healthcare, Sweden). Whole blood drawn into sodium heparin blood collection tubes was diluted 3x with phosphate-buffered saline (PBS) without calcium or magnesium (Lonza, Walkersville, Maryland). Diluted cell suspensions were layered on Leucosep tubes and centrifuged for 15 min at 800 x g at room temperature (RT). Interphases containing PBMCs were harvested, washed with PBS, and centrifuged for 10 min at 250 g at RT before further processing. PBMC were processed for scRNAseq analysis using the 10x Genomics platform (10x Genomics, Pleasanton, CA). Sample processing for single-cell RNA-seq was done using Chromium Single Cell 3’ Library and Gel bead kit v2 (PN-120237) following manufacturer’s user guide (CG00052, 10x Genomics, Pleasanton, CA). cDNA and libraries were prepared following manufacturer’s user guide (10x Genomics). cDNA amplification and indexed libraries were prepared using 12 and 14 cycles of PCR, respectively. Libraries were profiled, quantified, and sequenced as described above (5′ single-cell gene expression libraries).

Seurat (version 3.0) was used to perform basic quality control on the raw 50 GEX matrices output from Cell Ranger 2.2.0. The Cell Ranger Single Cell Software Suite v.2.2.1 was used to perform sample de-multiplexing, alignment, filtering, and UMI (i.e., universal molecular identifier) counting (https://support.10xgenomics.com/single-cell-gene expression/software/pipelines/latest/what-is-cell-ranger). The data for each respective subpopulation were aggregated for direct comparison of single cell transcriptomes. Then, gene dispersion analysis implemented in Seurat was used to select highly variable genes, preserving genes with logarithmic mean expression between 0.0 and 3.0 and with logarithmic dispersion less than 0.5. Seurat (version 3.0) was used to analyze the PBMC GEX data. Genes with detected expression in at least five cells, and cells with at least ten genes detected were used. The first 20 principal components were used for clustering (resolution = 0.6) and for UMAP visualization. Clusters were identified based on genes that are enriched in a specific cluster. Immunophenotyping of PBMCs was inferred from the annotation of cluster-specific genes; Total T cells (*CD3D*, *CD3E*), CD8^+^ T cells (*CD3E*, *CD8A, CD8B*), B cells (*CD79A*), CD14 monocytes (*CD14*), and NK cells (*NKG7*-positive and *CD3E*-negative).

Differential gene expression analysis of CD8^+^ T cells from IL-6-high (plasma IL-6 >10 pg/mL) versus IL-6-low patients used raw counts of the samples and was performed by edgeR in R (version 2.13.0) using the generalized linear model workflow described in the edgeR manual. First, the sequencing reads for duplicate sequencing libraries were combined to produce a single set of sequencing reads for each sample, and the raw read counts for each gene were used to produce a DGEList object in edgeR. Genes were only included if they were represented by at least one read in all of the samples. The calcNormFactors() function was used to account for differences in the library size for each sample, and an experimental design model consisting of the batch and HS status was established. The functions estimateCommonDisp() and estimateTagwiseDisp() were used to estimate dispersion. Following this, differential expression was tested using the exact test based on qCML methods. The Benjamini-Hochberg correction was used with a false discovery cut-off of 0.05. Reactome pathway enrichment analysis was used to identify processes associated with differentially expressed genes.^71^

#### Software versions related to clinical data analysis

Computational analysis was performed using Cell Ranger software (10x Genomics, Pleasanton, CA) version 2.2.0, Perl version 5.18.4, R version 3.6.0, and the following packages and versions in R for analysis: Seurat, 3.0.0; edgeR, 3.26.0; cluster, 2.0.8; dynamicTreeCut, 1.63-1; UMAP, WGCNA, 1.66; and survival, 2.42-6. Figures and tables were generated using the following packages and versions in R: RColorBrewer, 1.1-2; ggplot2, 3.1.1; gridExtra, 2.3; ComplexHeatmap, 2.0.0; superheat, 1.0.0; colorspace, 1.3-2; dplyr, 0.7.8; and data for external datasets were obtained using GenomicDataCommons, 1.4.3; GEOquery, 2.48.0. The above R packages depended secondarily on the following support packages: Matrix, 1.2-17; Biobase, 2.40.0; BiocGenerics, 0.26.0; cowplot, 0.9.3; DDRTree, 0.1.5; edgeR, 2.13.0; irlba, 2.3.2; limma, 3.38.2; magrittr, 1.5; Matrix, 1.2-15; ranger, 0.10.1; and VGAM, 1.0-6.

#### IL6 *in situ* hybridization

For the detection of *IL6* mRNA expression in RCC tumors, *in situ* hybridization was performed on 4μm thick formalin-fixed, paraffin-embedded tissue sections mounted on glass slides. The process was automated on the Leica BOND Rx platform (Buffalo Grove, IL). A 20 base-pair probe to the target region of *IL6* (2-1082) was used (Advanced Cell Diagnostics, Inc., Newark, CA). Tissue sections were pre-treated with heat and protease before hybridization with oligonucleotide probes. Detection and amplification were performed with the RNAscope 2.5 LSx Reagent Kit in Red (Advanced Cell Diagnostics, Inc., Newark, CA). Tumor sections were analyzed by a qualified pathologist and considered *IL6* positive if at least 1% of either tumor cell area or stromal area showed *IL6* stain. Epithelial and stromal cells were distinguished based on a combination of features and also evaluation of an adjacent, hematoxylin/eosin-stained section. Tumor cells in Figure 1D are characterized by larger nuclei with variable shape and outline and a more "open" chromatin pattern. Stromal cells show smaller and condensed nuclei. Tumor cells also have more abundant cytoplasm compared to stromal cells and show a more cohesive growth pattern of trabecular or acinar shapes.

#### *In vivo* tumor studies

Tumor cell lines in log-phase growth were centrifuged, washed with Hank’s balanced salt solution (HBSS), counted, and resuspended in 50% HBSS and 50% Matrigel (BD Biosciences; San Jose, CA). EMT6 cells were inoculated in the left #5 mammary fat pad (1 × 10^5^ cells in 100 μL of HBSS/Matrigel mixture). Mice were inoculated subcutaneously in the right flank with 1 × 10^5^ CT26 cells, 1 × 10^6^ MC38 cells, or 2 × 10^6^ E.G7-OVA cells in 100 μL of HBSS/Matrigel mixture. When tumors reached a volume of 130–230 mm^3^ (approximately 8 days after inoculation), animals were distributed into treatment groups such that variance in tumor sizes between treatment groups was minimized. Mice were treated with isotype control antibodies, anti-PD-L1 (mouse IgG1 clone 6E11, 10 mg/kg first dose followed by 5 mg/kg thereafter), anti-IL6R (mouse IgG2a clone MR16-1, 15 mg/kg), or a combination of anti-PD-L1 and anti-IL6R. Anti-PD-L1, anti-IL6R, and isotype control antibodies were produced in-house and were free of endotoxin contamination. For EMT6, CT26, and MC38 studies, mice were euthanized after 10–11 days (after 3 doses of treatment) and tumors collected for flow cytometry analysis. For E.G7-OVA studies, tumors were evaluated one week after adoptive transfer of 1 × 10^6^ OT-I CD8^+^ T cells. For therapeutic efficacy studies, antibodies were administered 2 times per week for 3 weeks (intravenously for the first dose and intraperitoneally thereafter). Tumors were measured 2 times per week using digital calipers, and tumor volumes calculated using the modified ellipsoid formula, 1/2 x (length x width^2^). When tumor volumes fell below 32 mm^3^ (lower limit of detection) they were considered a complete response (CR; 100% tumor growth inhibition). Tumors that regressed to less than 50% of starting volume but eventually recurred were considered partial responders (PR), and tumors that never regressed were considered to be progressive disease (PD). Disease progression was defined as a 5x increase in tumor volume. Mice were euthanized if tumors ulcerated or volumes exceeded 2000 mm^3^. Early euthanasia of some mice due to tumor ulceration contributed to some variability in final sample sizes of immune pharmacodynamic studies. No mice met criteria for euthanasia due to body weight loss or adverse clinical signs.

#### DEC-OVA immunization

Naive OT-I T cells were isolated from spleens and lymph nodes of OT-I or OT-I.IL6R.ko mice (both CD90.1+) by first mashing through 70 μm pore filters using the sterile blunt end of a plunger from a 1 mL syringe. Naive CD8^+^ T cells were then isolated using the EasySep Mouse Naive CD8^+^ T cell Isolation Kit (STEMCELL Technologies, Cambridge, MA). Cells were resuspended at 1 × 10^7^ cells/mL in sterile HBSS and 1 × 10^6^ cells (0.1 mL) were injected intravenously via the lateral tail vein into wild type C57BL/6J (CD90.2) recipient mice bearing EG.7-OVA tumors. The next day mice were injected intravenously with a mixture of 50 μg/kg DEC-OVA (ovalbumin fused to anti-DEC205 antibody; produced in-house) and 2.5 mg/kg anti-CD40 antibody (produced in-house; clone FGK4.5).

#### EMT6 cell proliferation *in vitro*

EMT6 cell responsiveness to IL-6 (10 ng/mL) and hyper-IL-6 (20 ng/mL) was quantified using a p-STAT3 (Y705) electrochemiluminescence assay after 15 min of stimulation (Meso Scale Diagnostics, Rockville, MD). Cell proliferation in the presence or absence of hyper-IL-6 (20 ng/mL) or anti-IL6R antibody (40 μg/mL) was determined by seeding cells into flat-bottom 96-well plates and tracking confluence over time using IncuCyte live-cell imaging (Essen BioScience, Ann Arbor, MI).

#### Tissue preparation and flow cytometry

Tumors were weighed, minced, and enzymatically digested using a cocktail of dispase (Life Technologies, Carlsbad, CA), collagenase P, and DNaseI (Roche, Penzberg, Germany) for 45 min at 37°C to obtain a single-cell suspension. Cells were counted using a Vi-CELL XR (Beckman Coulter, Brea, CA). Cell suspensions were passed through 100 μm pore filters to remove clumps and debris. For analysis of cytokine expression, cells were re-stimulated *ex vivo* for 3 h at 37°C in T cell stimulation media composed as follows: RPMI 1640 medium with 10% FBS (Hyclone, Waltham, MA), 100 U/mL penicillin/100 μg/mL streptomycin (Gibco, Thermo Fisher Scientific, Waltham, MA), 55 μM β-mercaptoethanol (Gibco, Thermo Fisher Scientific, Waltham, MA), 2 mM L-glutamine (Gibco, Thermo Fisher Scientific, Waltham, MA), 1 mM sodium pyruvate (Gibco, Thermo Fisher Scientific, Waltham, MA), 0.1 mM non-essential amino acids (Gibco, Thermo Fisher Scientific, Waltham, MA), 10 mM HEPES (Gibco, Thermo Fisher Scientific, Waltham, MA), and 1x Cell Stimulation Cocktail with protein transport inhibitors (containing phorbol 12-myristate 13-acetate (PMA), ionomycin, brefeldin A, and monensin; eBioscience, Thermo Fisher Scientific, Waltham, MA). For cell staining, cells were first incubated with anti-CD16/CD32 Fc block (5 μg/mL; BD Biosciences, San Jose, CA; clone 2.4G2) and LIVE/DEAD Fixable dead cell stain (efluor780; Invitrogen, Carlsbad, CA) in PBS for 20 min at 4–8°C. Cells were then washed and stained with combinations of the following antibodies: CD45-BV510 (2 μg/mL; BD Biosciences, San Jose, CA; clone 30-F11), Thy1.2-efluor450 (2 μg/mL; eBioscience, Thermo Fisher Scientific, Waltham, MA; clone 53-2.1), Thy1.2-alexafluor700 (5 μg/mL; BioLegend, San Diego, CA; clone 53-2.1), Thy1.1-alexafluor488 (2.5 μg/mL; BioLegend, San Diego, CA; clone OX-7), CD3ε-PE/Cy5.5 (2 μg/mL; eBioscience, Thermo Fisher Scientific, Waltham, MA; clone 145-2C11), CD4-BUV395 (2 μg/mL; BD Biosciences, San Jose, CA, clone GK1.5), CD4-BV785 (2 μg/mL; BioLegend, San Diego, CA; clone GK1.5), CD8a-BB515 (2 μg/mL; BD Biosciences, San Jose, CA, clone 53-6.7), CD8a-PE (2 μg/mL; BioLegend, San Diego, CA; clone 53-6.7), CD8a-BUV737 (2 μg/mL; BD Biosciences, San Jose, CA; clone 53-6.7), CD11b-alexafluor700 (5 μg/mL; BioLegend, San Diego, CA; clone M1/70), Gr1-PE-Cy5.5 (1 μg/mL; eBioscience, Thermo Fisher Scientific, Waltham, MA; clone RB6-8C5), CD11c-PE/Dazzle594 (2 μg/mL; BioLegend, San Diego, CA; clone N418), MHCII (I-A/I-E)-FITC (2.5 μg/mL; eBioscience, Thermo Fisher Scientific, Waltham, MA; clone M5/114.15.2), CD64-PE/Cy7 (2 μg/mL; BioLegend, San Diego, CA; clone X54-5/7.1), CD169-PE/Cy7 (2 μg/mL; BioLegend, San Diego, CA; clone 3D6.112), B220-BUV737 (2 μg/mL; BD Biosciences, San Jose, CA; clone RA3-6B2), IL6R-PE (2 μg/mL; BioLegend, San Diego, CA; clone D7715A7), and gp130-PE (2 μg/mL; BioLegend, San Diego, CA). Cells were stained for 20 min at 4–8°C.

Staining patterns used to define cell subsets were as follows: CD8^+^ T cells, Thy1^+^ CD3ε^+^ CD8a^+^ CD4^−^ CD11b^−^; CD4^+^ Tconv cells, Thy1^+^ CD3ε^+^ CD8a^−^ CD4^+^ Foxp3^−^ CD11b^−^; Treg, Thy1^+^ CD3ε^+^ CD8a^−^ CD4^+^ Foxp3^+^ CD11b^−^; macrophages, CD11b^+^ CD64^+^ Gr1^−^; monocytes/Mo-MDSC, CD11b^+^ Gr1^int^ CD64^int^; neutrophils/Gr-MDSC, CD11b^+^ Gr1^high^ CD64^−^; B cells, Thy1^−^ CD11b^−^ B220^+^ MHCII^+^ CD4^−^; plasmacytoid DC, Thy1^−^ CD11b^−^ B220^+^ MHCII^int^ CD4^+^ CD11c^+^; classical DC1, Thy1^−^ B220^−^ CD64/CD169^−^ CD11c^+^ MHCII^+^ CD11b^−^ CD8a^+^; classical DC2, Thy1^−^ B220^−^ CD64/CD169^−^ CD11c^+^ MHCII^+^ CD11b^+^ CD8a^−^.

For analysis of intracellular proteins, surface-stained cells were fixed and permeabilized with the eBioscience Foxp3/Transcription Factor staining buffer set (Thermo Fisher Scientific, Waltham, MA). Cells were then stained for 30–60 min at 4–8°C with combinations of the following antibodies in 1x permeabilization buffer: Foxp3-efluor450 (2 μg/mL; eBioscience, Thermo Fisher Scientific, Waltham, MA; clone FJK-16s), GzmB-Pacific Blue (1 μg/mL; BioLegend, San Diego, CA; clone GB11), TNF-PE (1 μg/mL; BioLegend, San Diego, CA; clone MP6-XT22), IFNγ-PE/Dazzle594 (0.67 μg/mL; BioLegend, San Diego, CA; clone XMG1.2), and IL-17A-BV785 (1 μg/mL; BioLegend, San Diego, CA; clone TC11-18H10).

Flow cytometry data were collected with a BD LSRFortessa or BD FACSymphony analyzer (BD Biosciences, San Jose, CA) and analyzed using FlowJo software (Version 10.5, FlowJo LLC, Ashland, OR). Cell sorting was performed on a BD Fusion/S6 (BD Biosciences, San Jose, CA).

#### T cell activation *ex vivo*

Mouse spleens and/or lymph nodes were isolated and mashed through 70 μm pore filters. For standard peptide activation of OT-I CD8^+^ T cells, 0.2 million splenocytes were seeded in Falcon flat bottom 96 well plates (Corning Life Sciences, Corning, NY) and pulsed with 100 ng/mL SIINFEKL peptide (AnaSpec, Fremont, CA), allowing splenic antigen-presenting cells to present both peptide and PD-L1 to T cells. After 2 days, cells were analyzed or transitioned to T cell media (without SIINFEKL) containing 10 ng/mL recombinant human IL-2 and incubated for 3–4 days before use in cytotoxicity assays or re-stimulation with anti-CD3/CD28 antibodies. For flow cytometry analysis of cytokine expression, Brefeldin A (eBioscience, Thermo Fisher Scientific, Waltham, MA) was added 4 h before staining.

For polyclonal T cell activation, bulk splenocytes or CD8^+^ T cells isolated using the EasySep CD8^+^ T cell Isolation Kit (STEMCELL Technologies, Cambridge, MA) were plated in T cell media containing 10 ng/mL human IL-2 at 0.2 million cells per well in Falcon flat bottom 96 well plates (Corning Life Sciences, Corning, NY) coated overnight with 5 μg/mL anti-CD3 antibody (BD Biosciences, San Jose, CA, clone 145-2C11) and 2.5 μg/mL anti-CD28 antibody (BD Biosciences, San Jose, CA, clone 37.51). Cells were labeled in some experiments with Cell Trace Violet-421 (Molecular Probes, Thermo Fisher Scientific, Waltham, MA). In some assays, T cells were activated in the presence of recombinant mouse IL-6 (10 ng/mL; R&D Systems, Minneapolis, MN), mouse hyper-IL-6 (20 ng/mL; R&D Systems, Minneapolis, MN), isotype control mouse IgG2a antibody (5 μg/mL), mouse IgG2a anti-IL6R antibody (5 μg/mL; clone MR16-1), or mouse IgG1 anti-PD-L1 (5 μg/mL; clone 6E11). CRISPR/Cas9-mediated deletion of *Batf* in primary T cells was performed according to a previously published protocol^72^ using the following gRNA targeting exon 1 (GGGGGTACCTGTTTGCCAG; IDT, San Diego, CA). BATF was detected in western blots using anti-BATF clone D7C5 (Cell Signaling Technology, Danvers, MA).

For human T cell activation, naive human CD8^+^ T cells were isolated from buffy coats using the EasySep Human Naive CD8^+^ T cell Isolation Kit (STEMCELL Technologies, Cambridge, MA), and plated on 96-well flat-bottom plates pre-coated with 10 μg/mL anti-CD3 (clone OKT3, Thermo Fisher Scientific, Waltham, MA) in T cell medium containing 1 μg/mL soluble anti-CD28 (clone CD28.2, BD Biosciences, San Jose, CA) and 10 ng/mL recombinant human IL-2 (R&D Systems, Minneapolis, MN). Recombinant human IL-6 (R&D Systems, Minneapolis, MN) was added to some wells at 10 ng/mL. Cells were re-stimulated with PMA/ionomycin before analysis.

#### T cell cytotoxicity assays

OT-I CD8^+^ T cells were activated with SIINFEKL peptide (AnaSpec, Fremont, CA) as described above in the presence or absence of recombinant mouse IL-6 (10 ng/mL) or hyper-IL-6 (20 ng/mL) for 5–6 days. MC38-GFP cells (plated at 5,000 cells per well in 96-well flat-bottom plates) were pulsed with 10 ng/mL SIINFEKL peptide for 1 h at 37°C and washed with PBS, at which time T cells were added in complete T cell medium. MC38 cell death was quantified over time using IncuCyte Live Cell Analysis (Essen Bioscience, Ann Arbor, MI).

#### Bulk RNA-seq analysis of mouse T cells

OT-I CD8^+^ T cells were activated with SIINFEKL peptide as described above. Viable CD8^+^ T cells were sorted to >99% purity on day 7 using a BD FACS Aria Fusion cell sorter (BD Biosciences, San Jose, CA). RNA was extracted using the RNEasy mini kit (Qiagen, Germantown, MD). 0.1 μg of total RNA was used for library preparation using TruSeq Stranded Total RNA Library Prep Kit (Illumina, San Diego, CA). Libraries were multiplexed and sequenced on Illumina HiSeq4000 (Illumina, San Diego, CA) to generate 30 million single-end 50 base pair reads. Data were analyzed using HTSeqGenie in BioConductor as follows: first, reads with low nucleotide qualities (70% of bases with quality <23) or rRNA and adapter contamination were removed. The reads that passed were then aligned to the reference genome GRCh38.p10 using GSNAP (Wu et al., 2016). Alignments of the reads that were reported by GSNAP as “uniquely mapping” were used for subsequent analysis. Gene expression levels were quantified as Reads Per Kilobase of exon model per Million mapped reads normalized by size factor (nRPKM), defined as number of reads aligning to a gene in a sample/(total number of uniquely mapped reads for that sample x gene length x size factor). Principal components analysis (PCA) and generation of differential expression heatmaps were performed using Partek Flow version 8.0.19.0710. Differential gene expression analysis was performed using voom from the limma R package.^73^

#### RNA extraction and qRT-PCR

Cells were lysed in RLT buffer and homogenized by pipetting. RNA was isolated using RNEasy Mini or Micro kits (Qiagen, Germantown, MD) followed by reverse transcription using the iScript cDNA synthesis kit (Biorad, Hercules, CA). Quantitative RT−qPCR was performed using Taqman assays (Life Technologies, Carlsbad, CA) according to manufacturer instructions and a ViiA7 384-well real-time PCR detection system (Applied Biosystems, Waltham, MA). All expression levels were normalized to an internal housekeeping gene (*Rpl19*) and calculated as 2^∧^−(CTHK−CTgene).

#### Western blotting

Cell pellets were lysed in RIPA buffer with protease and phosphatase inhibitors (Roche, Penzberg, Germany) at 4°C for 20 min. Supernatants were obtained after high-speed centrifugation and protein concentration measured using the BCA assay (Thermo Fisher Scientific, Waltham, MA). Lysates were denatured with reducing sample buffer and dithiothreitol (Invitrogen, Waltham, MA) at 95°C for 10 min. Proteins were separated by sodium dodecyl sulphate-polyacrylamide gel electrophoresis in a NuPAGE 4–12% gradient Bis-Tris gel and analyzed by western blotting with antibodies against phospho-STAT3 (clone D3A7, Cell Signaling Technology, Danvers, MA), total STAT3 (clone D1B2J, Cell Signaling Technology), BATF (clone D7C5, Cell Signaling Technology), and β-actin-HRP (Sigma-Aldrich, St Louis, MO).

### Quantification and statistical analysis

The Kaplan-Meier method was used to estimate the probability of OS. For OS analysis, surviving patients were censored at the time of the last contact. The hazard ratios and 95% confidence intervals for OS were estimated by a Cox regression model. Cox proportional hazards and linear regression model was performed to conduct multivariate analysis (co-variates are specified in figure legends).

Statistical calculations were performed using GraphPad software or R. Statistical details for specific experiments including the tests used and value/definition of *n* are provided in figure legends. Box-and-whisker plots represent median, quartiles, and maximum/minimum values. Bar charts and error bars indicate means and SEM. *In vitro* assays were performed with 3–4 technical replicates per condition. Sample sizes for *in vivo* mouse studies were based on the number of mice routinely needed to establish statistical significance based on variability within study arms. Each data point represents either a technical replicate (in the case of *in vitro* studies) or a biological replicate (individual mouse from *in vivo* studies). p-values and FDR values < 0.05 were considered in all analyses to be statistically significant.

### Additional resources

Clinical trial information for IMmotion150 (NCT01984242): https://clinicaltrials.gov/ct2/show/NCT01984242.

Clinical trial information for PCD4989g (NCT01375842): https://www.clinicaltrials.gov/ct2/show/NCT01375842.

Clinical trial information for IMvigor210 (NCT02951767 and NCT02108652): https://clinicaltrials.gov/ct2/show/NCT02951767 and https://clinicaltrials.gov/ct2/show/NCT02108652.

Clinical trial information for IMvigor211 (NCT02302807): https://clinicaltrials.gov/ct2/show/NCT02302807.

## Data Availability

• Qualified researchers may request access to individual patient-level data through the clinical study data request platform (http://www.clinicalstudydatarequest.com). Further details on Roche’s criteria for eligible studies are available here (https://clinicalstudydatarequest.com/Study-Sponsors/Study-Sponsors-Roche.aspx). For further details on Roche’s Global Policy on the Sharing of Clinical Information and how to request access to related clinical study documents, see here (http://www.roche.com/research_and_development/who_we_are_how_we_work/clinical_trials/our_commitment_to_data_sharing.htm). • Human tumor gene expression data for IMmotion150 are publicly available at the European Genome-Phenome Archive (EGA) under accession number EGA: EGAS00001004387 (Yuen et al., *Nature Medicine*, 2020). • Raw counts of human PBMC scRNA-seq data analyzed in this study have been submitted to the EGA with accession numbers EGA: EGAS00001004451, EGAS00001004458. • Raw and processed count matrix data from RNA-seq analysis of mouse T cells have been submitted to Gene Expression Omnibus as Super-Series GEO: GSE199048. • This paper does not report original code. • Any additional information required to reanalyze the data reported in this paper is available from the lead contact upon request.

## Supplemental information

Document S1. Figures S1–S5 and Tables S1–S3

Table S4. Reactome pathway analysis of differentially expressed genes associated with low or high (>10 pg/mL) plasma IL-6 in circulating CD8^+^ T cells from both UC and RCC patients, related to Figures 2I–2K

Table S5. Reactome pathway analysis of differentially expressed protein-coding genes regulated by IL-6 in CD8^+^ T cells *in vitro* and *in vivo*, related to Figures 3H, 5H, and 5I

Document S2. Article plus supplemental information
