# Supplementary material for: CD8+ T cell-intrinsic IL-6 signaling promotes resistance to anti-PD-L1 immunotherapy
Source: Cell Rep Med. 2023 Jan 3;4(1):100878. doi: 10.1016/j.xcrm.2022.100878 (PMC9873827; doi:10.1016/j.xcrm.2022.100878)
Supplement: Document S1. Figures S1–S5 and Tables S1–S3 [file mmc1.pdf]

Cell Reports Medicine, Volume *4*

Supplemental information

CD8$^+$ T cell-intrinsic IL-6 signaling promotes resistance to anti-PD-L1 immunotherapy

Mahrukh A. Huseni, Lifen Wang, Joanna E. Klementowicz, Kobe Yuen, Beatrice Breart, Christine Orr, Li-fen Liu, Yijin Li, Vinita Gupta, Congfen Li, Deepali Rishipathak, Jing Peng, Yasin Şenbabaoğlu, Zora Modrusan, Shilpa Keerthivasan, Shravan Madireddi, Ying-Jiun Chen, Eleanor J. Fraser, Ning Leng, Habib Hamidi, Hartmut Koeppen, James Ziai, Kenji Hashimoto, Marcella Fassò, Patrick Williams, David F. McDermott, Jonathan E. Rosenberg, Thomas Powles, Leisha A. Emens, Priti S. Hegde, Ira Mellman, Shannon J. Turley, Mark S. Wilson, Sanjeev Mariathasan, Luciana Molinero, Mark Merchant, and Nathaniel R. West

Supplementary Figure 1

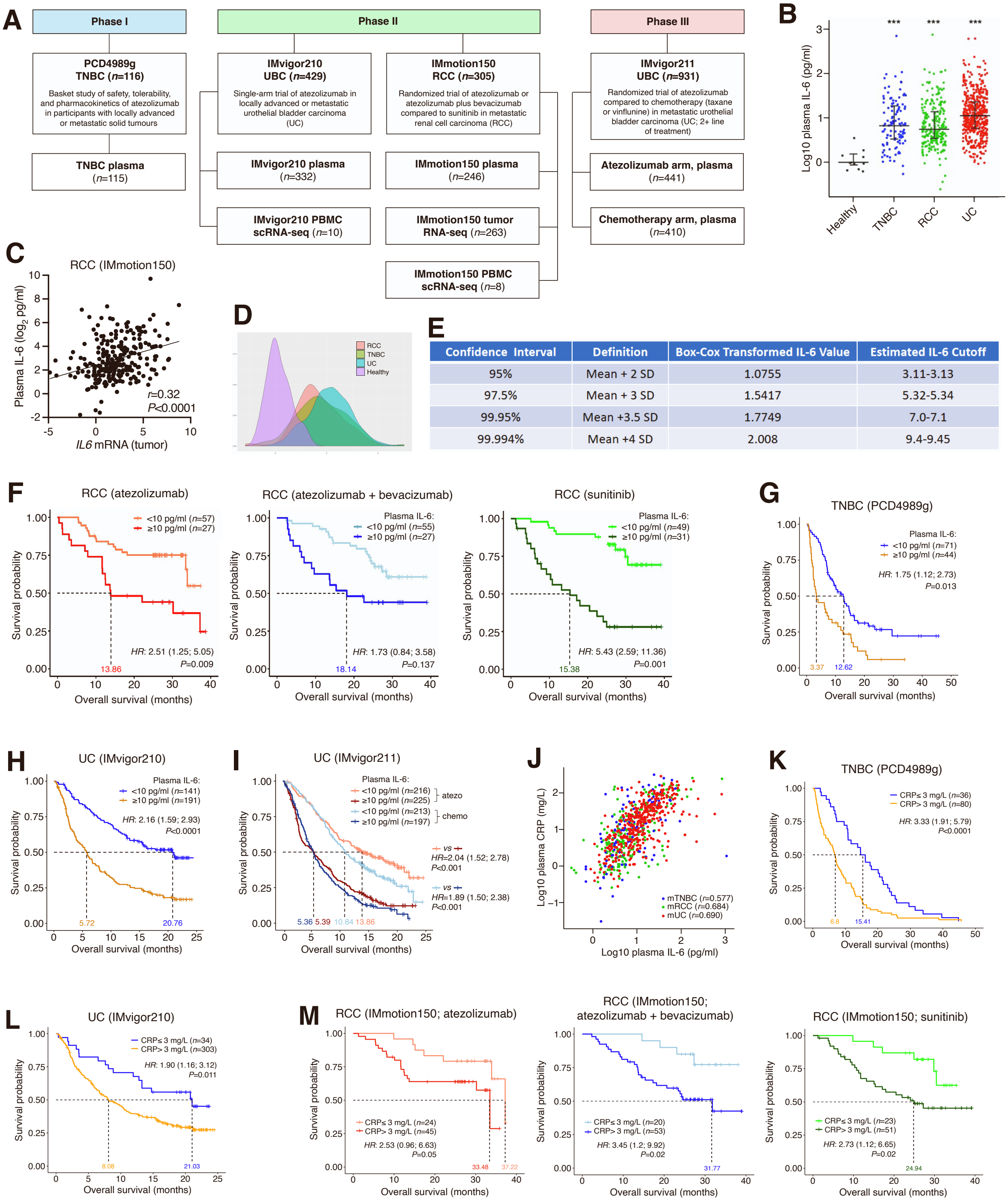

Confidence Interval	Definition	Box-Cox Transformed IL-6 Value	Estimated IL-6 Cutoff
95%	Mean + 2 SD	1.0755	3.11-3.13
97.5%	Mean + 3 SD	1.5417	5.32-5.34
99.95%	Mean +3.5 SD	1.7749	7.0-7.1
99.994%	Mean +4 SD	2.008	9.4-9.45

Legend on following page

Supplementary Figure 1. Plasma IL-6 and CRP associate with poor response to atezolizumab (anti-PD-L1) in multiple human cancers (related to Figure 1).

(**A**) Flowchart showing number of intent-to-treat patients in PCD4989g (TNBC cohort), IMvigor210, IMvigor211, and IMmotion150, as well as the numbers of patients whose plasma, bulk tumor RNAseq, or PBMC single-cell RNAseq samples were included for analysis.

(**B**) Plasma IL-6 concentrations in healthy individuals compared to patients with TNBC (P=1.87x10^{-6}), RCC (P=4.93x10^{-7}), or UC (P=1.35x10^{-7}), compared using two-sided Mann-Whitney *U*-tests with Benjamini-Hochberg correction.

(**C**) Pearson correlation of patient-matched tumor *IL6* mRNA expression (derived from RNAseq) and plasma IL-6 protein concentration in patients from IMmotion150.

(**D**) Distribution of plasma IL-6 in healthy adults and patients with the indicated cancer types.

(**E**) Plasma IL-6 concentrations were transformed into normality using Box-Cox transformation, and values were derived at the stated standard deviations and confidence intervals relative to the plasma IL-6 distribution of healthy adults. A concentration of ≥10 pg/ml (>4 standard deviations from the healthy control mean) was chosen for downstream analyses as the definition of high plasma IL-6.

(**F**–**I**) Association of high plasma IL-6 with poor OS in IMmotion150 (**F**), the TNBC cohort of PCD4989g (**G**), IMvigor210 (**H**) and IMvigor211 (**I**).

(**J**) Pearson correlation of plasma IL-6 and plasma CRP in patients with TNBC, UC, or RCC.

(**K**–**M**) Association of high plasma CRP (>3 mg/L) with poor OS in the TNBC cohort of PCD4989g (**K**), IMvigor210 (**L**), and IMmotion150 (**M**).

In all survival analyses, *HR* (+/− 95% CI) and *P*-values were corrected in multivariate analysis using the following co-variates: ECOG (Eastern Cooperative Oncology Group) performance status, liver metastasis, and line of therapy for TNBC; ECOG performance status and presence of liver metastasis for UC; and MSKCC (Memorial Sloan Kettering Cancer Centre) prognostic risk score, previous nephrectomy, and liver metastasis for RCC.

Supplementary Figure 2

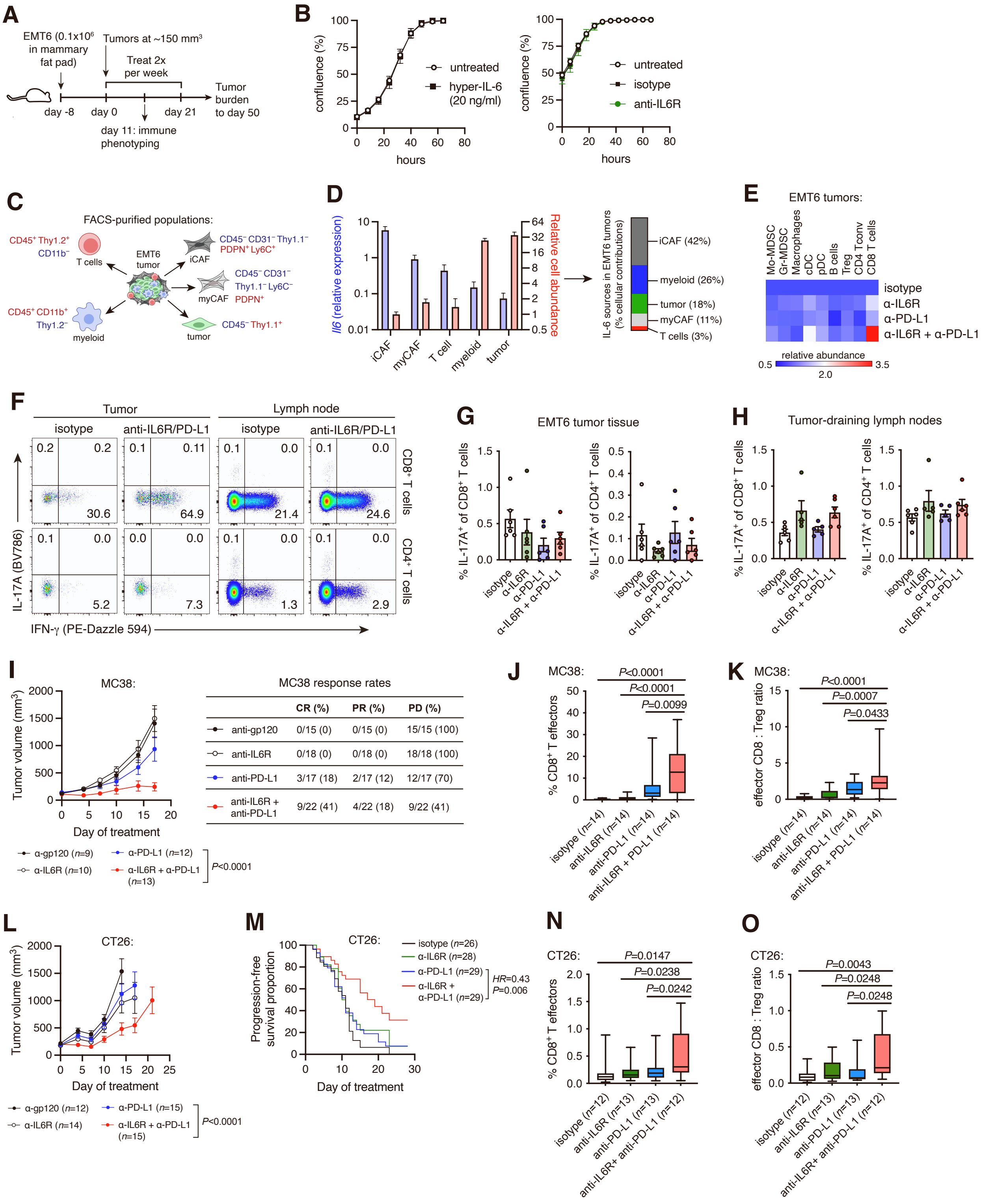

MC38 response rates

	CR (%)	PR (%)	PD (%)
anti-gp120	0/15 (0)	0/15 (0)	15/15 (100)
anti-IL6R	0/18 (0)	0/18 (0)	18/18 (100)
anti-PD-L1	3/17 (18)	2/17 (12)	12/17 (70)
anti-IL6R + anti-PD-L1	9/22 (41)	4/22 (18)	9/22 (41)

Legend on following page

Supplementary Figure 2. IL-6 inhibits anti-PD-L1 efficacy and effector differentiation of $CD8^+$ T cells (related to Figure 2).

(**A**) Blockade of IL6R and PD-L1 in the orthotopic EMT6 mammary tumor model. Tumors reached 150 mm^3 before randomization to treatment groups. Mice were treated with anti-IL6R and/or anti-PD-L1 antibodies twice weekly for 3 weeks for long-term tumor control studies, and for 10–11 days for analysis of tumor immune infiltrates.

(**B**) *In vitro* proliferation of EMT6 cells in the presence of hyper-IL-6 or anti-IL6R antibody, measured by Incucyte confluence assay (*n*=4 technical replicates ± s.e.m. per condition). EMT6 cells do not respond to IL-6 due to lack of IL6R expression, but are sensitive to hyper-IL-6 (a fusion protein of IL-6 and the soluble form of IL6R).

(**C**) FACS-sorting strategy to isolate major cell populations from EMT6 tumors.

(**D**) RT-qPCR analysis of *IL6* mRNA expression in cells isolated from untreated EMT6 tumors (from *n*=3 mice) as indicated in panel **C** (*n*=3 biological replicates). Blue bars indicate relative *IL6* expression (normalized to *Hprt*) in each cell type, while salmon bars indicate the relative abundance of each cell population as determined by flow cytometry (with the rarest cell type, iCAFs, set at 1). *IL6* expression and relative cell abundance were combined to estimate the percentage of overall *IL6* expression attributable to each cell type in the tumor, displayed as a stacked bar chart.

(**E**) Relative abundance of leukocyte populations in EMT6 tumors after treatment. Heatmap indicates mean fold change relative to isotype control, from *n*=16–17 mice pooled from three studies. Combination treatment caused a significant (*P*=0.0003) increase in $CD8^+$ T cells relative anti-PD-L1 alone, but no significant changes were observed for other cell types.

(**F**–**H**) Analysis of Th17 (IL-$17A^+$ $CD4^+$ T cells) and Tc17 (IL-$17A^+$ $CD8^+$ T cells) cells in EMT6 tumor tissue and draining lymph nodes (*n*=5–6 mice per group). Representative flow cytometry plots are shown in panel **F**. IL-$17A^+$ cell frequencies are provided for tumor and lymph nodes in panels **G** and **H**, respectively. Data are representative of three independent experiments.

(**I**–**K**) Anti-PD-L1 and/or anti-IL6R treatment of established (150 mm^3) subcutaneous MC38 tumors.

(**I**) MC38 tumor growth after treatment randomization. Anti-PD-L1 and combination treatment groups compared using two-way ANOVA (*P*-value reported for treatment effect). Rates of CR (complete tumor regression), PR (tumor regression of >50% followed by re-growth), and PD (persistent growth or regression <50%) are provided in the accompanying table (containing data pooled from two studies).

(**J**) Frequency of IFN-γ^+ $CD8^+$ T cells among tumor-infiltrating $CD45^+$ cells isolated from MC38 tumors after 10 days of treatment. Biological replicates are pooled from two studies. Groups compared using one-way ANOVA with Holm-Sidak's multiple comparisons test.

(**K**) Ratio of IFN-γ^+ $CD8^+$ T cells to $Foxp3^+$ $CD4^+$ Treg cells in MC38 tumors after 10 days of treatment. Biological replicates are pooled from two studies. Groups compared using one-way ANOVA with Holm-Sidak's multiple comparisons test.

(**L**–**O**) Anti-PD-L1 and/or anti-IL6R treatment of established (150 mm^3) subcutaneous CT26 tumors.

(**L**) CT26 tumor growth after treatment randomization (*n*=5–6 mice per group). Anti-PD-L1 and combination treatment groups compared using two-way ANOVA (*P*-value reported for treatment effect). Unlike MC38, complete tumor regression was not observed in this tumor model.

(**M**) Time-to-progression Kaplan-Meier analysis (5x increase in tumor volume) of CT26 tumors, starting from time of treatment. Anti-PD-L1 and combination groups compared using log-rank test. Biological replicates are pooled from three studies.

(**N**) Frequency of polyfunctional $CD8^+$ T cells among tumor-infiltrating $CD45^+$ cells isolated from CT26 tumors after 10 days of treatment. Data are pooled from two studies. Groups compared using one-way ANOVA with Holm-Sidak's multiple comparisons test.

(**O**) Ratio of effector $CD8^+$ T cells to $Foxp3^+$ $CD4^+$ Treg cells in CT26 tumors after 10 days of treatment. Data are pooled from two studies. Groups compared using one-way ANOVA with Holm-Sidak's multiple comparisons test.

Supplementary Figure 3

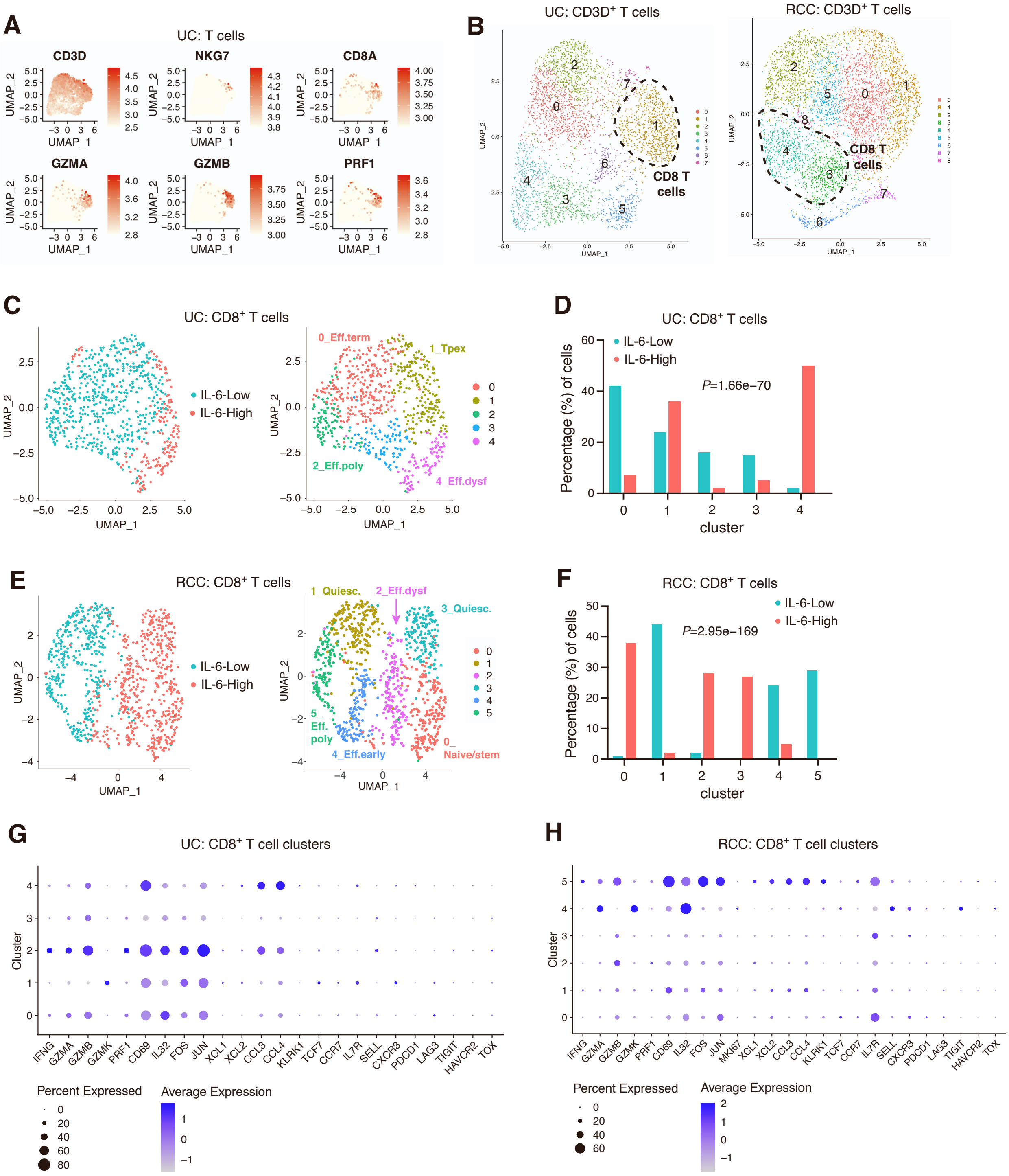

Legend on following page

Supplementary Figure 3. Single-cell RNA-seq analysis of CD8$^+$ T cells from patients with high or low plasma IL-6 (related to Figure 2I–K).

(**A**, **B**) For samples from both UC and RCC cohorts, PBMC were clustered using UMAP analysis to identify total T cells (**A**), which were then sub-clustered to identify CD8$^+$ T cells (**B**) based on co-expression of markers such as *CD8A*, *GZMB*, *NKG7*, and *PRF1*.

(**C**, **E**) CD8$^+$ T cells from UC (**C**) and RCC (**E**) samples were further UMAP-clustered to identify transcriptionally distinct subpopulations. 5 subclusters were identified for UC-derived samples, and 6 subclusters for RCC-derived samples. Proposed cluster identities are indicated as follows: *Eff.poly*, polyfunctional effectors; *Eff.early*, early differentiated effectors; *Eff.dysf*, dysfunctional effectors; *Naïve/stem*, naive/central memory/stem-like cells; *Quiesc*., quiescent with high expression of ribosomal genes and low expression of effector markers; *Tpex*, T progenitor exhausted.

(**D**, **F**) Frequency distributions of CD8$^+$ T cell subclusters among cells derived from UC (**D**) or RCC (**F**) patients with high or low plasma IL-6. Cluster distributions were compared using Chi-square analysis.

(**G**, **H**) Dot plots representing expression of key genes associated with effector function, naïve/memory-like states, or exhaustion among CD8$^+$ T cell subclusters from UC (**G**) and RCC (**H**) samples. UC cluster 2 and RCC cluster 5 are defined as polyfunctional effector cells based on co-expression of multiple effector markers, including *IFNG* and *GZMB*.

Supplementary Figure 4

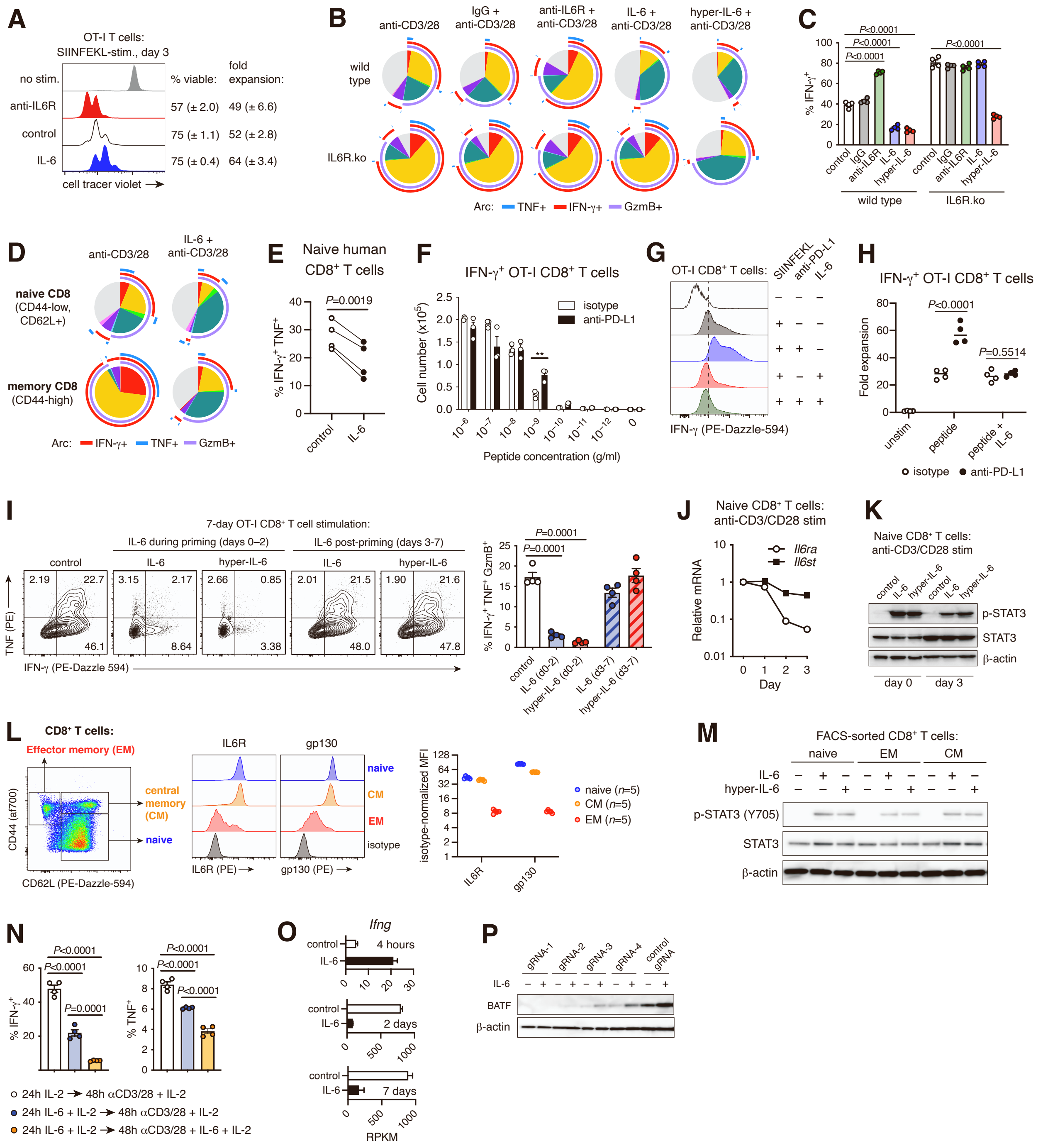

Supplementary Figure 4. Inhibition of CD8+ T cell function by IL-6 (related to Figures 3–4).

(**A**) Proliferation of OT-I CD8+ T cells following stimulation of bulk splenocytes with SIINFEKL peptide and IL-6 or anti-IL6R antibody. Mean % viable cells and fold expansion values (± s.e.m.) are provided for day 3, based on *n*=4 technical replicates per group. Data are representative of three experiments.

(**B**) Boolean analysis (represented as SPICE plots) of IFN-γ, TNF, and GzmB expression in polyclonally activated $CD8^+$ T cells. Splenocytes from IL6R.ko mice or WT littermates were cultured with plate-bound anti-CD3/CD28 antibodies in the presence of isotype control antibody, anti-IL6R antibody, IL-6, or hyper-IL-6. $CD8^+$ T cells were analyzed after 3 days. Data represent 3 independent experiments. Colored arcs represent the proportion of cells expressing the indicated factor. Overlapping arcs indicate factor co-expression. Pie segments are colored uniquely to highlight different co-expression phenotypes (e.g. red = IFN-γ^+ $GzmB^+$ TNF^+; yellow = IFN-γ^+ $GzmB^+$ TNF^-; gray = IFN-γ^- $GzmB^-$ TNF^-, etc).

(**C**) IFN-γ^+ frequencies among $CD8^+$ T cells from panel **B**. Groups compared using one-way ANOVA with Tukey's multiple comparisons test.

(**D**) Boolean analysis of IFN-γ, TNF, and GzmB expression in FACS-sorted naïve or memory $CD8^+$ T cells from WT C57BL/6J mice, activated for 3 days with anti-CD3/CD28 antibodies +/− IL-6. Data are representative of two independent experiments.

(**E**) Activation of human naïve $CD8^+$ T cells isolated from PBMC of n=4 healthy donors. T cells were activated with plate-bound anti-CD3 and soluble anti-CD28 antibody, with or without 10 ng/ml recombinant human IL-6. Cells were restimulated on day 5 with PMA/ionomycin and analyzed by flow cytometry for expression of IFN-γ and TNF. Groups compared using paired t-test.

(**F**–**H**) Inhibition of anti-PD-L1 effects by IL-6 in an *ex vivo* OT-I T cell activation assay based on peptide-stimulation of bulk splenocytes. $CD8^+$ T cell expansion and phenotype was assessed by flow cytometry. Data points represent technical replicates.

(**F**) SIINFEKL peptide was titrated in 10-fold dilutions to optimize detection of anti-PD-L1-driven enhancement of $CD8^+$ T cell activation. Groups compared by t-test; $^{**}P<0.01$.

(**G**) Flow cytometry analysis of IFN-γ expression after activation with 1 ng/ml SIINFEKL in the presence of isotype control antibody, anti-PD-L1 antibody, IL-6, or combined anti-PD-L1 and IL-6.

(**H**) Relative expansion of IFN-γ^+ OT-I $CD8^+$ T cells activated as described in panel **G**. Groups compared by t-test; data represent two independent experiments.

(**I**) OT-I splenocytes were cultured with SIINFEKL peptide in the presence or absence of IL-6 or hyper-IL-6 for 2 days, at which time IL-6 was removed and cells were maintained in medium supplemented with IL-2 for 4 days. Cells were restimulated with anti-CD3/CD28 for 1 day and $CD8^+$ T cells were analyzed by flow cytometry on day 7. Alternatively, cells were activated for the first 2 days with peptide only, and IL-6 or hyper-IL-6 was added from days 3-7. Representative contour plots of IFN-γ and TNF expression are shown, in addition to summarized frequencies of polyfunctional cells among $CD8^+$ T cells. Groups compared using one-way ANOVA with Holm-Sidak's multiple comparisons test. Data are representative of two experiments.

(**J**) Naïve $CD8^+$ T cells from WT C57BL/6J mice were activated with anti-CD3/CD28 antibodies and cells were collected daily for analysis of *Il6ra* and *Il6st* (gp130) expression by qRT-PCR (normalized to *Rpl19*). Data points indicate mean (± s.e.m.) of technical triplicates.

(**K**) Naïve $CD8^+$ T cells from WT C57BL/6J mice were stimulated with IL-6 or hyper-IL-6 for 15 minutes, either at resting state or after 3 days of anti-CD3/CD28 stimulation, and cells were harvested for analysis of STAT3 phosphorylation (Y705) by western blot.

(**L**) Flow cytometry analysis of IL6R and gp130 expression on naïve, CM, and EM $CD8^+$ T cells from healthy C57BL/6J mice. Representative histograms are shown in the center panel, and quantification of protein expression (relative to isotype control) in the right panel. Data points indicate biological replicates.

(**M**) Naïve, CM, or EM $CD8^+$ T cells from WT C57BL/6J mice were isolated by FACS, rested in culture for 1 hour without TCR or cytokine stimulation, and then treated with IL-6 or hyper-IL-6 for 15 minutes. Cells were lysed and analyzed for STAT3 phosphorylation (Y705) by western blot.

(**N**) WT $CD8^+$ T cells (C57BL/6J) were activated in one of the following 3 ways: standard anti-CD3/CD28 stimulation after culture with IL-2 for 1 day; culture with IL-6 + IL-2 for 1 day prior to anti-CD3/CD28 stimulation, at which time IL-6 was withdrawn; or culture with IL-6 + IL-2 for 1 day prior to anti-CD3/CD28, with continuous IL-6 stimulation for the duration of the experiment. IFN-γ and TNF expression was assessed by flow cytometry on

day 3 following anti-CD3/CD28 stimulation. Groups of *n*=4 technical replicates were compared using one-way ANOVA with Tukey's multiple comparisons test; data represent two independent experiments.

(**O**) *Ifng* mRNA expression in $CD8^+$ T cells with or without IL-6 stimulation at 4h, 2d, or 7d following TCR stimulation. Bars indicate mean (± s.e.m.) of *n*=3 technical replicates. *Associated with Fig. 4A*.

(**P**) Generation of BATF CRISPR-ko cells from primary $CD8^+$ T cells using electroporation of Cas9-gRNA complexes. Cells were electroporated two days prior to anti-CD3/CD28 stimulation and maintained in medium containing 5 ng/ml recombinant IL-7. BATF deletion was assessed by western blot 2 days after anti-CD3/CD28 stimulation +/− IL-6. Out of 4 BATF-targeting gRNA sequences tested, gRNA-1 was selected for downstream functional studies. *Associated with Fig. 4G*.

Supplementary Figure 5

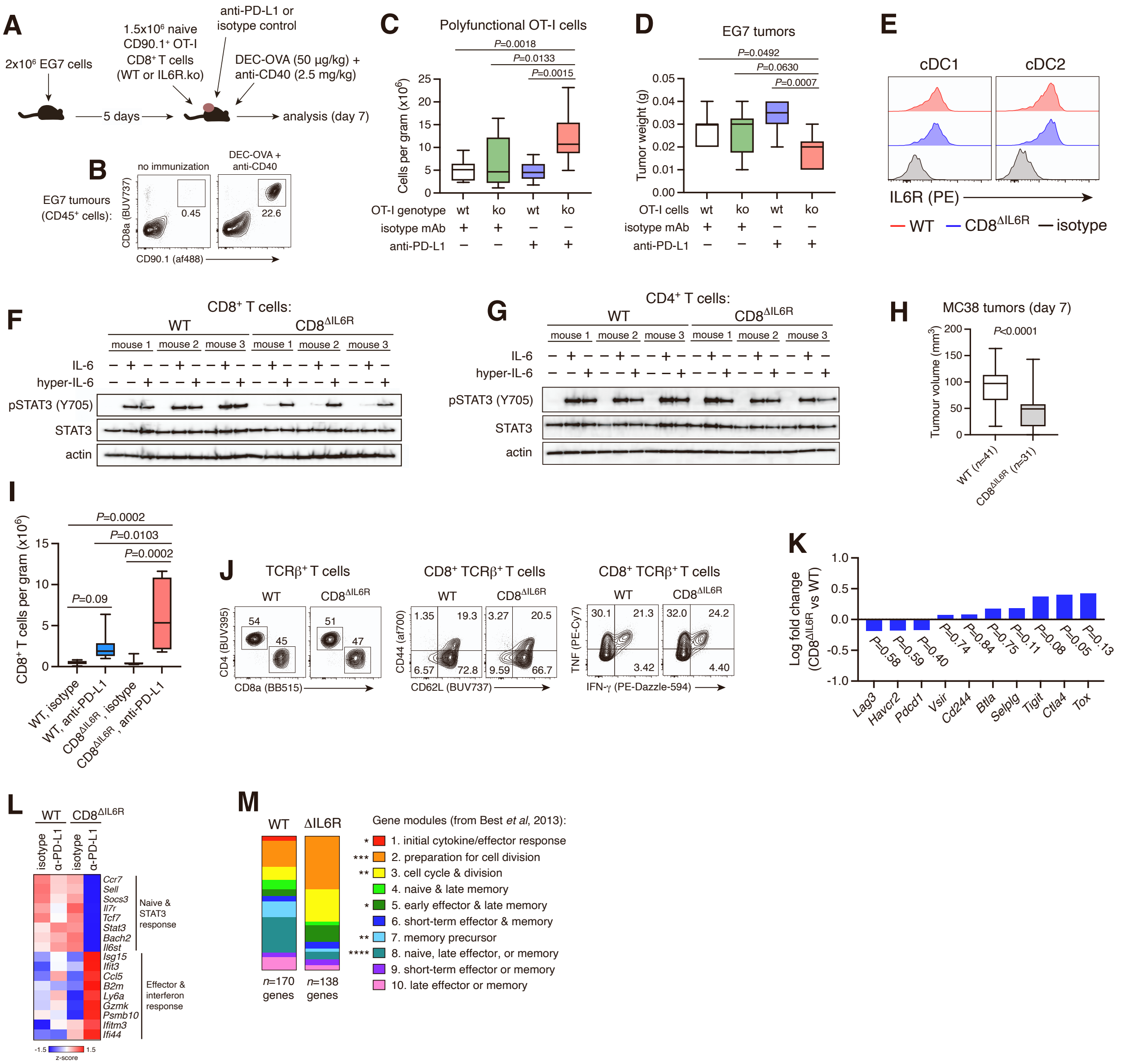

Supplementary Figure 5. Suppression of anti-PD-L1 response *via* CD8$^+$ T cell-intrinsic IL-6 signaling (related to Figure 5).

(**A**) Adoptive transfer and *in vivo* activation of WT or IL6R.ko OT-I CD8$^+$ T cells in mice bearing established EG7 tumors. All treatments were initiated on the same day. Isotype/anti-PD-L1 treatments were administered again 4 days later.

(**B**) Impact of DEC-OVA (ovalbumin fused to anti-DEC205 antibody) + anti-CD40 immunization on activation of CD90.1 (Thy1.1)$^+$ OT-I cells in EG7 tumor-bearing mice. OT-I cells are primed ineffectively toward EG7 tumors in the absence of additional immunization with ovalbumin. Cells are gated on total tumor-infiltrating CD45$^+$ cells.

(**C**, **D**) Abundance of polyfunctional (IFN-γ^+ GzmB$^+$ TNF$^+$) OT-I CD8$^+$ T cells normalized to tumor weight (**C**), and tumor masses (**D**) after one week of treatment. Data are from n=9–10 mice per group, compared using one-way ANOVA with Holm-Sidak's multiple comparisons test. Data are representative of three experiments.

(**E**) IL6R expression on lymph node-derived cDC1 (CD11b$^-$ CD8a$^+$ CD11c$^+$ MHC-II$^+$) and cDC2 (CD11b$^+$ CD8a$^-$ CD11c$^+$ MHC-II$^+$) from CD8$^{\Delta IL6R}$ mice or WT littermates.

(**F**, **G**) Western blot analysis of STAT3 activation in splenic CD8$^+$ T cells (**F**) or CD4$^+$ T cells (**G**) from healthy CD8$^{\Delta IL6R}$ mice or WT littermates. Cells were stimulated *ex vivo* for 20 minutes with 10 ng/ml IL-6 or 20 ng/ml hyper-IL-6.

(**H**) MC38 tumor volumes after one week of growth in untreated CD8$^{\Delta IL6R}$ mice or WT littermate controls. Groups compared by Mann-Whitney *U*-test.

(**I**) Total CD8$^+$ T cell abundance (normalized to tumor weight) in MC38 tumors of CD8$^{\Delta IL6R}$ mice or WT littermates after one week of anti-PD-L1 or isotype control treatment. Data are from n=6–9 mice per group from one of two independent experiments; groups compared using one-way ANOVA with Holm-Sidak's multiple comparisons tests.

(**J**) Lymph node-derived CD8$^+$ T cell frequency and phenotype in CD8$^{\Delta IL6R}$ mice versus WT littermates. For cytokine analysis, cells were restimulated *ex vivo* with PMA/ionomycin for 2 hours. Plots are representative of 3 healthy female mice per genotype.

(**K**) RNAseq analysis of exhaustion-related markers in CD8$^+$ T cells sorted from MC38 tumors in CD8$^{\Delta IL6R}$ mice or WT littermates treated with anti-PD-L1.

(**L**) Expression of selected genes associated with naïve T cells, STAT3 response, and IFN response in CD8$^+$ T cells sorted from MC38 tumors in CD8$^{\Delta IL6R}$ mice or WT littermates treated with anti-PD-L1 or isotype control antibodies.

(**M**) Distribution of differentially expressed genes from RNAseq analysis of tumor-infiltrating CD8$^+$ T cells from anti-PD-L1-treated CD8$^{\Delta IL6R}$ mice or WT littermates among CD8$^+$ T cell differentiation modules 1–10 from Best *et al* (*Nat Immunol*, 2013; OT-I cells activated *in vivo* by infection with *Listeria*-OVA). *P<0.05, >0.01; **P<0.01, >0.001; ***P<0.001, >0.0001; ****P<0.0001 (Fisher's exact test).

Variable	IMmotion150 - Atezo Total N (%)	IL6 low N (%)	IL6 High N (%)	P value	IMmotion150 - Atezo+Bev Total N (%)	IL6 low N (%)	IL6 High N (%)	P value	IMmotion150 - Sunitinib Total N (%)	IL6 low N (%)	IL6 High N (%)	P value
**Age**												
All patients	84	57	27	NA	82	54	28	NA	80	49	31	NA
median age (years) (range)	62 (26-81)	63 (37-81)	61 (26-79)	NA	61 (35-87)	61 (35-87)	60 (48-74)	NA	62 (25-85)	61 (25-85)	62 (39-78)	NA
**Sex**												
Male	63 (75)	43 (75)	20 (74)		61 (74)	36 (67)	25 (89)		65 (81)	40 (82)	25 (81)	
Female	21 (25)	14 (25)	7 (26)	1	21 (26)	18 (33)	3 (11)	0.033	15 (19)	9 (18)	6 (19)	1
**Race**												
White	75 (89	49 (86)	26 (97)		72 (88)	48 (89)	24 (85)		73 (92)	43 (88)	30 (97)	
Black	3 (4)	3 (5)	0 (0)		2 (2)	1 (2)	1 (4)		1 (1)	1 (2)	0 (0)	
Asian	1 (1)	0 (0)	1 (3)		2 (2)	1 (2)	1 (4)		1 (1)	1 (2)	0 (0)	
Other	5 (6)	5 (9)	0 (0)	0.26	6 (7)	4 (7)	2 (7)	0.728	5 (6)	4 (8)	1 (3)	0.239
**Liver Mets**												
Yes	23 (27)	17 (30)	6 (22)		23 (28)	9 (17)	14 (50)		16 (20)	10 (21)	6 (19)	
No	61 (73)	40 (70)	21 (78)	0.625	59 (72)	45 (83)	14 (50)	0.004	64 (80)	39 (79)	25 (81)	1
**PNEPH**												
Yes	75 (89)	54 (95)	21 (78)		72 (88)	50 (93)	22 (79)		69 (86)	45 (92)	24 (77)	
No	9 (21)	3 (5)	6 (22)	0.027	10 (12)	4 (7)	6 (21)	0.083	11 (14)	4 (8)	7 (23)	0.1
**MSKCC**												
Favorable	21 (25)	18 (32)	3 (11)		21 (26)	18 (33)	3 (11)		12 (15)	8 (16)	4 (13)	
Intermediate	57 (68)	38 (67)	18 (67)		53 (64)	36 (67)	17 (61)		59 (73)	38 (78)	21 (68)	
Poor	6 (7)	1 (1)	6 (22)	0.05	8 (10)	0 (0)	8 (28)	0.005	9 (11)	3 (6)	6 (19)	0.758
**SLD**												
median (mm) (range)	55 (12-265)	49 (12-265)	93 (22-254)	0.043	73 (17-295)	67 (17-295)	91 (21-278)	0.031	57 (10-288)	52 (10-208)	86 (21-288)	0.042

Supplementary Table 1 (related to Figure 1). Demographic characteristics of RCC (renal cell carcinoma) patients in the IMmotion150 study. IL-6 status refers to plasma IL-6 (high, >10 pg/ml).

Variable	IMvigor210 Total N (%)	IL6 low N (%)	IL6 High N (%)	P value		IMvigor211-Atezo Total N (%)	IL6 low N (%)	IL6 High N (%)	P value	IMvigor211-Chemo Total N (%)	IL6 low N (%)	IL6 High N (%)	P value
**Age**					**Age**								
All patients	332	141	191	NA	All patients	441	216	225	NA	410	213	197	NA
median age (years) (range)	68 (32-91)	68 (42-89)	68 (32-91)	NA	median age (years) (range)	67 (33-88)	67 (33-88)	67 (39-88)	NA	67 (31-84)	68 (31-82)	67 (36-84)	NA
**Sex**					**Sex**								
Male	263 (80)	108 (77)	155 (81)		Male	336 (76)	157 (73)	179 (80)		321 (78)	164 (77)	157 (80)	
Female	69 (20)	33 (23)	36 (19)	0.34	Female	105 (24)	59 (27)	46 (20)	0.09	89 (22)	49 (23)	40 (20)	0.55
**Race**					**Race**								
White	302 (91)	125 (89)	177 (93)		White	315 (71)	148 (68)	167 (74)		291 (71)	151 (70)	140 (72)	
Black	9 (3)	5 (4)	4 (2)		Black	1 (1)	0 (0)	1 (1)		2 (1)	0 (0)	2 (1)	
Asian	8 (2)	4 (3)	4 (2)		Asian	62 (13)	31 (15)	31 (14)		51 (12)	24 (12)	27 (13)	
Other	13 (4)	7 (4)	6 (3)	0.246	Other	63 (14)	37 (17)	26 (11)	0.169	66 (16)	38 (18)	28 (14)	0.741
**Liver Mets**					**Liver Mets**								
Yes	93 (28)	32 (23)	61 (32)		Yes	128 (29)	55 (25)	73 (32)		107 (26)	34 (16)	73 (37)	
No	239 (72)	109 (73)	130 (68)	0.065	No	313 (71)	161 (75)	152 (68)	0.116	303 (74)	179 (84)	124 (63)	1.16E-06
**TMB**					**TMB**								
<10	152 (59)	66 (59)	86 (58)		<10	120 (55)	74 (59)	66 (59)		120 (53)	59 (50)	61 (51)	
>=10	107 (41)	45 (41)	62 (42)	0.899	>=10	98 (45)	52 (41)	46 (41)	1	108 (47)	59 (50)	49 (49)	0.429
**ECOG**					**ECOG**								
0	127 (38)	78 (55)	49 (26)		0	208 (47)	132 (61)	76 (34)		183 (45)	115 (54)	68 (35)	
1 or 2	205 (62)	63 (45)	142 (74)	2.63E-11	1 or 2	235 (53)	84 (39)	151 (66)	5.63E-09	227 (55)	98 (46)	129 (65)	1.01E-04
**SLD**					**SLD**								
median (mm) (range)	55 (10-309)	36 (10-54)	80 (55-309)	0.012	median (mm) (range)	160 (1-281)	161 (1-281)	160 (5-278)	0.03	167 (3-280)	169 (3-280)	160 (5-280)	0.041

Supplementary Table 2 (related to Figure 1). Demographic characteristics of UC (urothelial bladder cancer) patients in the IMvigor210 and IMvigor211 studies. IL-6 status refers to plasma IL-6 (high, >10 pg/ml).

	PCD4989g - TNBC			
Variable	Total N (%)	IL6 low N (%)	IL6 High N (%)	P value
**Age**				
All patients	115	71	44	NA
median age (years) (range)	52 (29-79)	52 (29-79)	53 (33-76)	NA
**Sex**				
Male	0	0	0	
Female	115 (100)	71 (100)	44 (100)	1
**Race**				
White	88 (77)	58 (82)	30 (68)	
Black	7 (6)	3 (4)	4 (9)	
Asian	6 (5)	2 (3)	4 (9)	
Other	14 (12)	8 (11)	6 (14)	0.116
**Liver Mets**				
Yes	30 (26)	12 (17)	18 (41)	
No	85 (74)	59 (83)	26 (59)	0.008
**ECOG**				
0	52 (45)	41 (58)	11 (25)	
1 or 2	63 (55)	30 (42)	33 (75)	<0.001
**SLD**				
median (mm) (range)	5 (1-31)	4 (1-19)	9 (1-31)	0.013

Supplementary Table 3 (related to Figure 1). Demographic characteristics of TNBC (triple-negative breast cancer) patients in the PCD4989g study. IL-6 status refers to plasma IL-6 (high, >10 pg/ml).
